# IoT-Based Sensor Data Fusion for Determining Optimality Degrees of Microclimate Parameters in Commercial Greenhouse Production of Tomato

**DOI:** 10.3390/s20226474

**Published:** 2020-11-12

**Authors:** Sayed Moin-eddin Rezvani, Hamid Zare Abyaneh, Redmond R. Shamshiri, Siva K. Balasundram, Volker Dworak, Mohsen Goodarzi, Muhammad Sultan, Benjamin Mahns

**Affiliations:** 1 Department of Irrigation and Drainage, Faculty of Agriculture, Bu-Ali Sina University, Hamedan 6517833131, Iran; m.rezvani@areeo.ac.ir; 2 Leibniz Institute for Agricultural Engineering and Bioeconomy, Max-Eyth-Allee 100, 14469 Potsdam-Bornim, Germany; rshamshiri@atb-potsdam.de (R.R.S.); vdworak@atb-potsdam.de (V.D.); BMahns@atb-potsdam.de (B.M.); 3 Department of Agriculture Technology, Faculty of Agriculture, University Putra Malaysia, Serdang 43400, Selangor, Malaysia; siva@upm.edu.my; 4 Department of Mechanical Engineering, Faculty of Engnirreing, Bu-Ali Sina University, Hamedan 65175461, Iran; m.goodarzi@basu.ac.ir; 5 Department of Agricultural Engineering, Bahauddin Zakariya University, Bosan Road, Multan 60800, Pakistan; muhammadsultan@bzu.edu.pk

**Keywords:** IoT monitoring, LoRa sensors, greenhouse, optimum microclimate, simulink, wireless communication

## Abstract

Optimum microclimate parameters, including air temperature (T), relative humidity (RH) and vapor pressure deficit (VPD) that are uniformly distributed inside greenhouse crop production systems are essential to prevent yield loss and fruit quality. The objective of this research was to determine the spatial and temporal variations in the microclimate data of a commercial greenhouse with tomato plants located in the mid-west of Iran. For this purpose, wireless sensor data fusion was incorporated with a membership function model called Optimality Degree (OptDeg) for real-time monitoring and dynamic assessment of T, RH and VPD in different light conditions and growth stages of tomato. This approach allows growers to have a simultaneous projection of raw data into a normalized index between 0 and 1. Custom-built hardware and software based on the concept of the Internet-of-Things, including Low-Power Wide-Area Network (LoRaWAN) transmitter nodes, a multi-channel LoRaWAN gateway and a web-based data monitoring dashboard were used for data collection, data processing and monitoring. The experimental approach consisted of the collection of meteorological data from the external environment by means of a weather station and via a grid of 20 wireless sensor nodes distributed in two horizontal planes at two different heights inside the greenhouse. Offline data processing for sensors calibration and model validation was carried in multiple MATLAB Simulink blocks. Preliminary results revealed a significant deviation of the microclimate parameters from optimal growth conditions for tomato cultivation due to the inaccurate timer-based heating and cooling control systems used in the greenhouse. The mean OptDeg of T, RH and VPD were 0.67, 0.94, 0.94 in January, 0.45, 0.36, 0.42 in June and 0.44, 0.0, 0.12 in July*,* respectively. An in-depth analysis of data revealed that averaged OptDeg values, as well as their spatial variations in the horizontal profile were closer to the plants’ comfort zone in the cold season as compared with those in the warm season. This was attributed to the use of heating systems in the cold season and the lack of automated cooling devices in the warm season. This study confirmed the applicability of using IoT sensors for real-time model-based assessment of greenhouse microclimate on a commercial scale. The presented IoT sensor node and the Simulink model provide growers with a better insight into interpreting crop growth environment. The outcome of this research contributes to the improvement of closed-field cultivation of tomato by providing an integrated decision-making framework that explores microclimate variation at different growth stages in the production season.

## 1. Introduction

In recent years, due to population growth, scarcity of water supplies and limitations of arable land, a significant investment in greenhouse products in Iran has taken place [1,2]. The extent of greenhouse grown vegetable crops, strawberry and herbal plants in Iran has grown from 5946 hectares in 2011 to 11,034 hectares in 2019 showing 85.6% increase [3,4]. The growing numbers of resources that are allocated to closed-field cultivation demands special attention in terms of monitoring and control for sustainability of the production. In specific, greenhouse growers need to be provided with digital tools including sensors, mobile apps and knowledge-based decision support systems in order to achieve a comfortable growth condition that maintains optimum microclimate parameters for their crops. This is particularly important in commercial scale cultivation under closed-field environments, where disease can be spread in a shorter time and production failures impose serious setbacks. In this regard, wireless sensors and remote monitoring-and-control instrumentation that benefits from the concept of the Internet-of-Things (IoT) have been deployed in smart farming to help growers stay competitive at the market [5,6]. Wireless sensing becomes more demanding in greenhouse applications due to their high flexibility and functionality for real-time monitoring. They can be placed anywhere and overcome cable wiring difficulties for the sensors and the LAN connection. Additionally, they are also flexible in time and can be moved by user experience. However, the major disadvantage of wireless sensor nodes is the repeated loss of connection even in mesh applications. The water in the high amount of biomass of the plants damps the radio signals and avoids communication distances over long ranges. This can be solved by using different techniques (that sometimes involve a huge amount of effort), including antennas with cable for higher positions, higher mesh density, multiple gateway nodes and higher output power. In general, it is a good practice to store all measurement data using devices that benefits from local memory (i.e., dataloggers with onboard SD card). Therefore, the asynchronous readout is enabled for the user and the data is not missed for the overall greenhouse model. This can be done by hand or by mobile gateways passing the sensor node, which is a good application for IoT monitoring in large scale commercial greenhouse crop production. 

The basic concept of IoT devices is the interaction between objects with a specific address that connects to the world wide web [7]. The collected data is loaded in a cloud and analyzed more deeply, more rapidly and at a lower cost, with confidence and efficiency [6]. However, in most studies, raw data are first collected via a wireless sensor network-based systems and are analyzed afterward [8,9]. A drawback of this approach is that because the collected data is not processed in real-time, they cannot immediately determine the temporal and spatial variations in the microclimate parameters, as well as their deviation from optimal conditions. Knowing the fact that microclimate homogeneity affects the quantitative and qualitative performance of the products and can reduces energy consumption in greenhouses [8,9], a lack of real-time data monitoring and data processing system can result an inhomogeneous control. It has also been mentioned in various published literature that an effective IoT-based framework should incorporate the use of wireless sensors and mobile applications for displaying, processing and analyzing data from remote locations using cloud services which in themselves provide new insights and recommendations for better decision-making [7,10,11]. In fact, the main impact of the use of IoT in agriculture is to achieve higher crop yields with less cost. It is believed that by 2050, IoT devices will potentially increase agricultural productivity by 70% by converting various hidden aspects of the fields into data [12].

The amount of published literatures that have explored the use of IoT devices and wireless sensors in agriculture and greenhouse production is huge. One of the most recent examples includes the work of Munoz et al. (2020) on the design of greenhouse production services with IoT-based cloud system incorporating historical data, current values, meteorological forecast, a weather model, a tomato production model and an irrigation model through a REST API (Representational State Transfer; Application Programming Interface) service [13]. Zamora introduced a smart farming IoT platform based on edge and cloud computing for soilless culture greenhouses. This IoT platform saved more than 30% water relative to regular open control [14]. Liao developed an IoT-based system for monitoring the microclimate and growth status of phalaenopsis in orchid greenhouses that had an image recognition algorithm to determine the effect of environmental factors on orchid leaf size [15]. Shamshiri et al. (2020) developed a platform to calculate the optimality degree in tomato greenhouse cultivation based on data collected with IoT-sensors [16,17]. Their real-time data processing framework can also provide the results of the optimality degree of microclimate and the simulation of yield models [16,17]. Similar studies have also explored the horizontal distribution and homogeneity of the greenhouses’ temperature and humidity using wireless sensors [18,19,20]. An important highlight is reported by Katsoulas et al. [19] stating that in most theoretical studies, the greenhouse microclimate is considered to be uniform whereas in actual conditions the microclimate variables vary. The homogeneity of greenhouse microclimate leads to the homogeneity and good quality of produce, the reduction of diseases, lesser use of fungicides and energy saving [8,9,21]. The heterogeneity of greenhouse microclimate can bring about a significant difference in yield, productivity and the development of diseases [19]. Katsoulas et al. [19] also observed that the greatest heterogeneity of horizontal distribution of temperature and humidity occurred during daytime in summer cultivation. Contour plots of air temperature and relative humidity showed a clear difference for these factors in both daily and seasonal periods. Balendonck et al. [22] investigated the temporal and spatial distribution of microclimate parameters using multiple wireless sensors in six greenhouses. Utilizing geostatistical analysis, they concluded that at least 9 sensors per hectare were necessary to examine the horizontal distribution of temperature and humidity and to identify humid and cold sections in the greenhouse and high-risk regions of fungal disease. Van Dam [23] described that the most important effects on the horizontal and vertical climatic heterogeneity are the uneven conversion of solar radiation to heat throughout the greenhouse, ventilation, entrance of cold air from one side and leaving the relatively warm air from the other side of the greenhouse. In addition, several studies have focused on determining the optimal microclimate for greenhouse tomatoes [16,24,25,26,27,28,29,30]. Shamshiri et al. [16,17,28,29,31] introduced different levels of optimal and marginal values of air temperature (T), relative humidity (RH) and vapor pressure deficit (VPD) for closed-field cultivation of tomato under different light condition and growth stages. An index called optimality degree [26,31] has been also introduced that translates raw microclimate parameters into a number between zero and one, indicating how close a parameter is to the optimal growth condition at a specific growth stage and light condition. 

The research gap in the studies on the spatial and temporal distribution in greenhouse microclimate lies along the real-time processing of the collected data by means of data loggers or wireless sensors. In addition, most of the previous studies are carried out in small scale research greenhouses [16,20,24,25,26,28,32] within which the description of greenhouse climatic conditions has been based on raw data and the percentage difference between greenhouse microclimate and optimal microclimate conditions, along with processed data, has not been discussed. Only a few studies have been conducted in large scale commercial greenhouses using wireless sensor networks [8,19] or by means of IoT software and cloud-based computing frameworks, in which processed microclimate data have been made available to the end user. Our research was motivated by the vast and rapid development of greenhouse cultivation in Iran and the need for a real-time assessment tool that could be deployed and used by the growers to address the effect of different design scenarios on the optimality degree of microclimate parameters. For this purpose, IoT sensor fusion was incorporated by means of low-powered wide area network (LPWAN) sensors in the greenhouse and data were transmitted directly to a private cloud server. The VPD along with the optimality degree of microclimate parameters were calculated in the cloud and analyzed. The study was performed in an asymmetric commercial greenhouse to bring the research results closer to reality. The general objective of the project was to increase food security and self-sustainability of greenhouse crop production in mid-west of Iran. For this purpose, our research aimed at utilizing wireless sensing and IoT monitoring technology for increasing productivity and profitability (i.e., high yields at low expenses) from commercial greenhouses and to keep indoor-cultivation competitive. Therefore, the specific objective of this paper was to determine the spatial and temporal distribution of air temperature, relative humidity, VPD and their associate optimality degrees in cold and warm seasons based on the tomato growth stages. 

## 2. Materials and Methods

### 2.1. Experimental Setup

**Figure 1:**
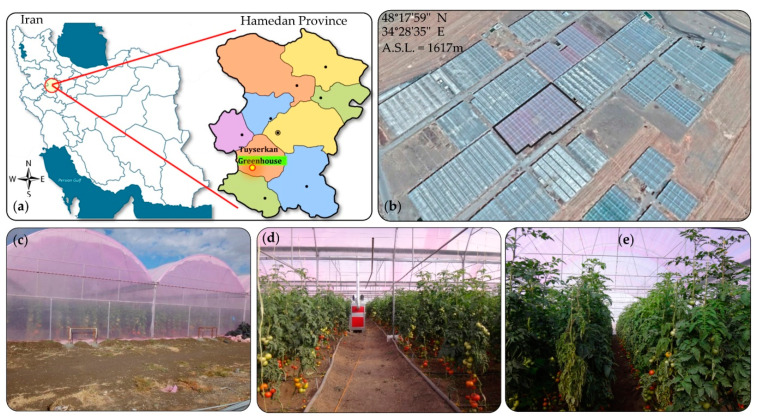
View of the experimental setup showing: (**a**) Locations of the greenhouse in the mid-west of Iran; (**b**) The aerial view of the greenhouse and its orientation; (**c**) outside view of the greenhouse in winter; (**d**) hot air heating system inside the greenhouse; and (**e**) view of tomato rows inside the greenhouse.

**Figure 2:**
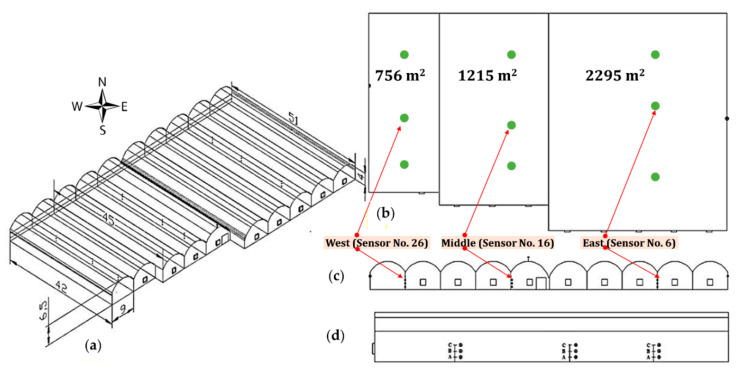
Schematic view of the greenhouse layout and the locations of wireless sensors: (**a**) perspective view of the greenhouse spans; (**b**) top view of the greenhouse spans with the green circles showing sensors locations; (**c**) front view of the greenhouse with sensors displayed by black dots; (**d**) right view of the greenhouse showing sensors at positions A = 0.60 m, B = 1.40 m, C = 2.20 m above the ground.

The greenhouse was located in the Tuyserkan region of the Hamedan province, located in the mid-west of Iran at longitude 48°17’59’’ N and latitude 34°28’35’’ E at 1617 m. A map of the research location, as well as the outside and inside view of the greenhouse under study is shown in Figure 1. Data collection was carried out between January 2018 and July 2018 in a Quonset commercial greenhouse with barrel vaults. The greenhouse roof had a double polyethylene covering of 200 µm external and a 60 µm internal layer. The floor area of the greenhouse was 4333 m^2^, with ten 9-m spans, an eave height of 4 m and a ridge height of 6.5 m. The greenhouse units were originally constructed as a two phase project, referred by growers as the old and new section, with the eave height difference of 2.75 m between the two phases. The distance between the old and new sections was 1.5 m which extended the total width of spans to 91.5 m. The shape of the greenhouse was asymmetric, consisting of three main units with different areas. The length of the greenhouse was 51 m in the old part including 5 spans to the area of 2295 m^2^. The new section was erected with three spans of 45 m in length with an area of 1215 m^2^ and 2 spans of 42 m in length and an area of 756 m^2^ as shown in Figure 2a,b.

The greenhouse facility was oriented to the north east–south west. During the first two data collections on 10 January 2018 and 17 January 2018, all three existing exhaust fans were turned off during the experiment. In the next two data collection period on 10 June 2018 and 29 July 2018, the number of exhaust fans was increased to 9. Two ventilation vents were provided on the eastern and western side-walls, a vent in the ending wall of the greenhouse (northern side) and a roof vent opening in the junction of the old and new sections. The lateral vents were located at a height of 3 m and could be opened to one meter. To prevent energy loss due to leakage or exchange with the air outside during the cold season, no roof ventilation was considered in the structure except one at the intersection of the new and old sections. The first tomato cultivation period was from the end of July to the end of January and the second cultivation was from mid-February to mid-July, 2018. A total of four data collections were carried out in winter (January), spring (June) and summer (July). During the winter and spring data collection period, the cultivated tomato crop was at the fruit formation and early fruiting stage (mature fruiting stage) and in the summer measurement, it was at the early growth stage. It should be noted that the cultivation was carried out on rows at 150 cm distance, in two plant rows at 36 × 37 cm. In order to determine the spatial variations in the microclimate data, the greenhouse was divided into three sub-sections and wireless sensors were installed laterally at three points as follow: one at the middle of the first section and the other two at one-third of the second and third sections. The locations of the sensors are shown schematically in Figure 2c,d.

### 2.2. Wireless Sensors and Data Collection

**Figure 3:**
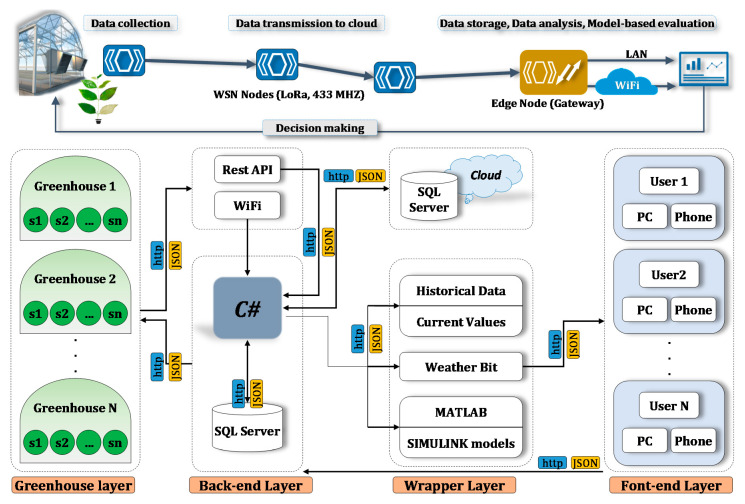
Schematic view of the modular network connectivity solution used for the data collection as described by References [13] and [16]. Picture is the courtesy of Adaptive AgroTech Consultancy International.

For the first three data collection, wireless sensors were placed in all 6 lateral points at the two heights of 1.4 and 2.2 m (canopy range). In the last data collection, due to younger plants, sensors were placed at a lower height of 0.6 and 2.2 m respectively. During the winter season, two vents in the east and west of the greenhouse were open between 8:30 and 16:30. In order to determine the effect of these vents a sensor was placed in the middle of each vent. In addition, in the third and fourth data measurement, in spring and summer, three wireless sensors were installed alongside the two side-wall vents and at the end-wall vent. The side-wall and end-wall vents were permanently open in the spring and summer and the ceiling vent were opened after the fans were switched off (the time-control fans operated between 8:30 and 20:30). For the purpose of data collection, the ADP-AgRoTech modular solar-powered LoRaWAN wireless solution with full network connectivity hardware and software packages was used (Adaptive AgroTech, KL, Malaysia). A schematic representation of the sensors nodes and gateway, as well as the architecture for data transmission and the wireless protocols is shown in Figure 3. For the sake of increasing accuracy and reliability, as well as minimizing data collection losses, hardware failures and wireless interruptions, microclimate data inside the greenhouse were collected using redundant instrumentation by simultaneous use of wireless nodes and data loggers that had onboard storage. The sensors nodes and IoT dashboard for real-time monitoring were customized for the research by the supplier. The details of the instrumentation can be found in Reference [16,17]. This integration enabled measuring of different environmental parameters including outside weather conditions, inside microclimate and light conditions, in a way that collected data from each sensor node could be pre-processed on the same node (distributed process) and only a final message were transferred to the gateway node and/or to the end user. The gateway node shown in Figure 3 was capable for over-the-air programming, which made possible re-programming it for data send-and-request. It should be noted that sensor nodes were also programmed to store, calculate and transmitted minute and hourly average microclimate data.

**Figure 4:**
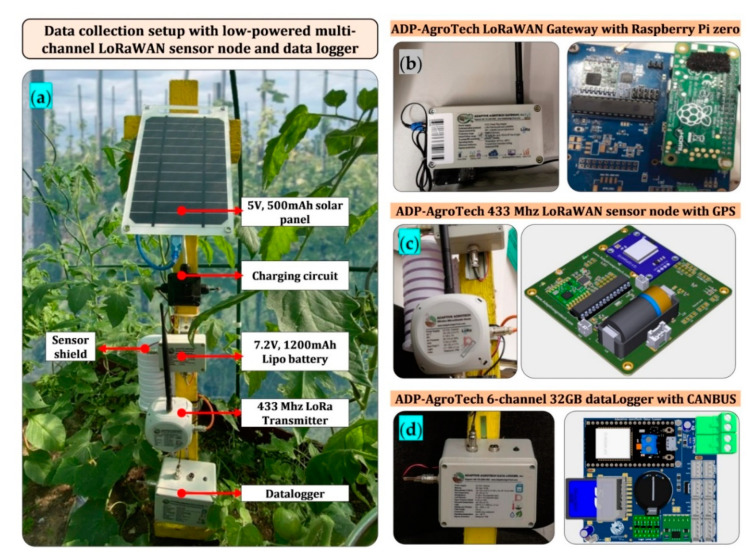
View of the wireless data acquisition system used in the experiment.

The housing cases for the instrumentation used in the study were waterproof and were all rated IP68. The data collection hardware was custom-designed to operate in harsh field condition, including high humidity of greenhouse environments. They were tested for operating in low and high temperature between −40 to 125 °C with a 0.01 °C and 1% air temperature and relative humidity resolution respectively. The accuracy and reliability of the sensors had been carried out and certified in an official calibration phase at the Leibniz Institute for Agricultural Engineering and Bioeconomy (Potsdam, Germany). Figure 4 shows the data collection setup including (a) the ADP-AgroTech multi-channel redundant wireless sensing platform, (b) the ADP-AgroTech LoRaWAN gateway, (c) the 433Mhz sensor node board with onboard GPS module and (d) the data logger unit with 32GB onboard storage and industrial CANBUS interface. The LoRaWAN gateway consisted of three main components, including a concentrator board that was connected to an antenna, a Raspberry Pi Zero single-board computer (Raspberry Pi Foundation, CA, UK) that made possible all the connections between the concentrator and the LoRaWAN backend and the codes that were custom-written to drive all the process. The gateway used the available Wi-Fi network inside the greenhouse office. It had a waterproof IP66 case, with GX16 aviation plug connector that was used to connect to external 5VDC power supply. The wireless sensor board utilized a 32-bit microprocessor integrated with a plug and sense probe and external solar-charged battery module. Wireless communication with the gateway node was realized via a LoRa SX1278 chip (Semtech, Camarillo, CA) rated at 433-470Mhz frequency mounted on the Dual-Core ESP32 240MHZ CP2102 (Espressif systems, Shanghai, China) that was interfaced with a Raspberry Pi zero board, and together were capable of covering a 2~10 km distance in rural areas. The coverage could be extended to over 50 km distance with repeaters. Microclimate sensor probe utilized a digital stainless steel DS1820 probe (Maxim Integrated, San Jose, CA), BlueDot BME280 + light sensor TSL2591 (ASM AG Inc, Premstaetten, Austria) and BMP280 (Bosch Sensortec Inc, Reutlingen, Germany) modules that were interfaced with ADP -WSN/LoRa, v4.0 board with onboard Wi-Fi and LoRa antenna that could be used as a stand-alone sensing unit for the direct transmission of data to cloud servers. It should be noted that the BME280 a combined digital humidity, pressure and temperature sensor based on proven sensing principles. Its small dimensions and low power consumption allowed the implementation in the greenhouse environment. In addition, the TSL2591 measurement range is between 188 μ Lux-88,000 Lux. Other specifications of the wireless transmitters and receiver are as follow, dimension: 100 × 100 × 1 mm, working voltage 3.3 V ~ 7 V, IP68 external power supply with two rechargeable 3.7 V 1200 mAh LiPo batteries, maximum admitted current: between 200 and 400 mA, recommended operating temperature: −40 ~ +85 °C, LoRa remote modem: 433/868/915MHz frequency (approx. 139 dBm high sensitivity, + 20 dBm, sensitivity receiver: up to–139 dBm). To measure the external climate variables, a customized position reporting system (APRS) weather station was installed on the greenhouse roof at an elevation of 7.5 m that collected outside Air T, RH, Air P and wind speed and solar radiation. This weather station included a SHT10 (Sensirion AG, Stäfa, Switzerland) sensor with an accuracy of 0.5 °C to 4.5%, an air pressure BMP180 (Bosch Sensortec, Inc., Reutlingen, Germany) sensor with an accuracy range of 0.12 hPa accuracy, a wind speed sensor VV1 (Nesa, Inc., Vidor, Italy) with an accuracy range of 0.1 m s^−1^, a TSL2591 lux meter, a precipitation sensor PL400 (Nesa, Inc., Vidor, Italy) with an accuracy range of 0.1 mm and a wind director.

### 2.3. IoT Monitoring and Data Analyses

**Figure 5:**
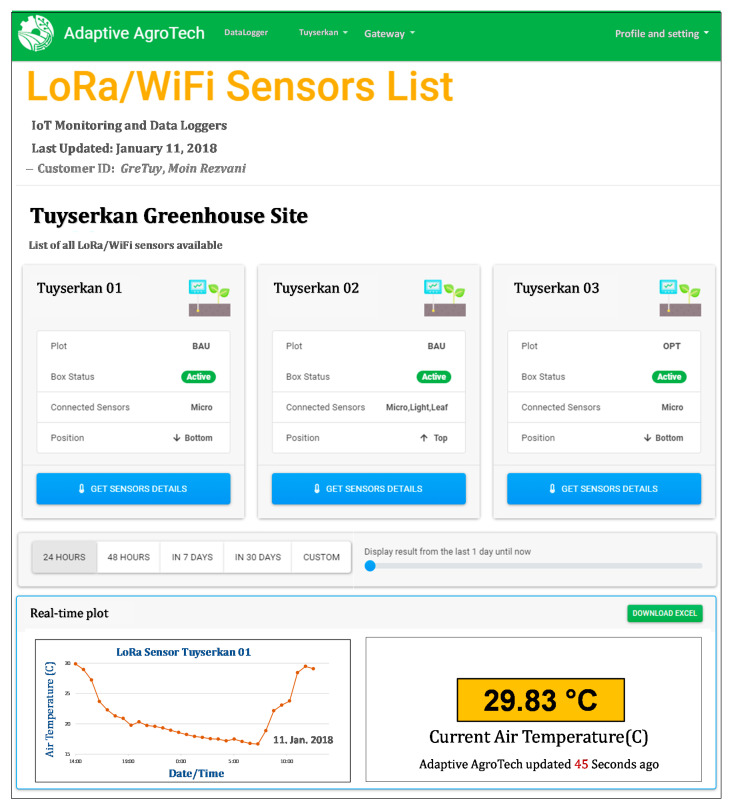
Screenshot of the Internet of Things (IoT) platform used for real-time data monitoring.

All measurement frequencies were set at 1Hz, with data stored on an onboard mini SD card (for the Datalogger board and the APRS weather station) and transferred to an open source secure cloud database (for the LoRa/Wi-Fi nodes). A customized IoT dashboard was designed for real-time monitoring of the measurements. Data visualization was made available via an android mobile app and via a secured webpage at http://iot.adaptiveagrotech.com/. The screenshot of the real-time data monitoring dashboard on the webpage is shown in Figure 5. 

**Figure 6:**
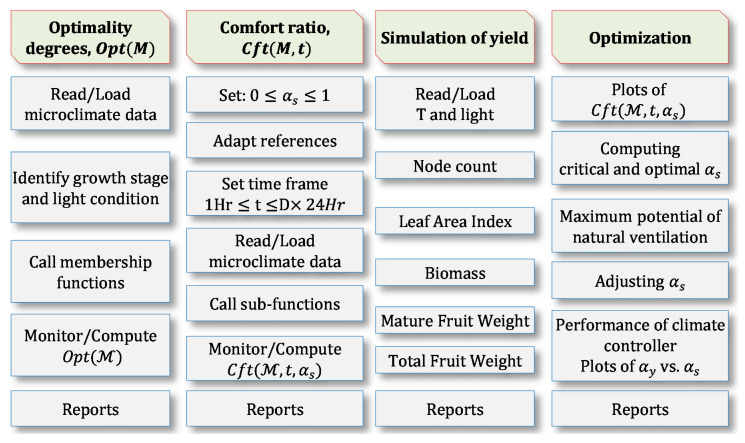
Screenshot of the adaptive management framework toolbox used for sensor fusion [17].

Data analysis was performed using the OptDeg toolbox of the Adaptive Analysis Framework (AAF) Software developed by Shamshiri et al. [16,17,28,29,31] as shown in Figure 6. The software is available as Simulink blocks in MATLAB (MathWorks, Natick, MA, USA) and has various tools for dynamic assessment of greenhouse microclimate data and simulating different scenario via an AAF approach. The software can be used for evaluating T, RH and VPD data as well as prediction of the expected yield in greenhouse using OptDeg, Cft and TOMGRO models, respectively. The software then creates databased that can be stored as Excel sheets or as text files directly from the hardware interfaces. The OptDeg was developed and used by Shamshiri et al. [16,26] that integrates a series of membership functions by taking into account the limitations in optimal air temperature and relative humidity at different growth stages and light conditions (sunny, cloudy, night) for tomato crop. The descriptions of the equations used in the AAF software are provided in Table 1, Table 2 and Table 3 for further references. In this model, tomato growth is divided into five growth stages including (i) germination and early growth with initial leaves, (ii) vegetative growth, (iii) flowering and fruit set, (iv) fruit formation and early fruiting and (v) mature fruiting. In this study, the growth stages are shown as GS1, GS2 and GS3–5. 

The OptDeg model was then used to demonstrate how close a microclimate measurement (ℳ: T, RH or VPD) is to their optimal references as required by the greenhouse crop at specific growth stage and climate condition. According to this model, a membership function for specific growth stage and light condition on the universe of discourse is defined as $Opt{\left(ℳ\right)}_{GS,\;\left(Light\right)}:ℳ\to \left[0,1\right]$, where ℳ: T,RH, VPD is the universe of discourse (input). In the other words, each ℳ readings in the greenhouse at time $t_{m,n}$, is mapped to a value between 0 and 1 that quantifies its optimality for tomato production. The two indexes m and n refer to specific minute and date of a measurement. In this model, an OptDeg equal to 1 refers to a potential yield with marketable value high quality fruit. For example, Opt(T)=1 is associated with T∈[24,27]℃ at the vegetative to mature fruiting growth stage during sun hours. The closer the optimality degree value is to 1, the closer the air temperature, relative humidity and VPD are ideal values for tomato cultivation at that stage of growth and light conditions. A detailed description of the membership functions and other mathematical models defining optimality degrees of T, RH and VPD and their shapes are fully explained in References [16,29,31]. In addition, the following equation was used to calculate VPD from air temperature T (°C) and relative humidity RH (%) which is widely used by greenhouse growers [17,29,31]:

(1) $$VPD\left(T,RH\right)=\left(1-\frac{\mathrm{RH}}{100}\right)\times 0.611\times \exp\left(17.27\times \frac{T}{T+237.3}\right)$$

The justification for calculating VPD is that it provides a better indication of the evaporation potential than RH and is capable of better reflecting how plant feels. It can be used to predict how close a plant production environment is to saturation in order to avoid condensation problems. For instance, in summer days, peak hours of high T and low level of RH, significantly increase VPD, leading to water stress. Large values of VPD, (approximately higher than 2 kPa) is a good indication that shows high transpiration rates which significantly increases in evapotranspiration (ET) demands and stomatal closure. Problems associated with calcium deficiency can be avoided by maintaining adequate transpiration. A comprehensive review of the optimal range of VPD and its application in greenhouse production can be found in Reference [29]. The SigmaPlot 12.3 software was used to filter raw data using the Negative Exponential method algorithm by Gaussian $e^{-x^{2}}$ weighting kernel and polynomial degree 2 to compute smoothed value method. Surfer 15.0 software was used to draw the contour maps of the microclimate parameters.

## 3. Results

The results are organized according to the data collections that were carried out on dates 10 January 2018, 17 January 2018, 10 June 2018 and 29 July 2018. In this paper, we refer to the first two data sets as the first and second measurements during the cold season and the second two data sets as the third and fourth measurement during the warm season. Temporal and spatial variations in the raw data sets and their corresponding optimality degree values were studied to provide results by means of two-dimensional and three-dimensional plots. In order to create plots of the 24-h variations, we selected three sensors that were located in the west (sensor ID. 26), in the middle (sensor ID. 16) and in the East (sensor ID. 6) sections of the greenhouse. These sensors were spaced at a distance of 9.0, 36.3 and 73.6 m from the western side of the greenhouse, at a height of 2.2 m, relative to the surface of the greenhouse at a distance from the western wall of the greenhouse equal to 378 (8.7%), 1580 (36.5%) and 3415 (78.8%) square meters. In order to prevent any interference among the charts, the graph of changing microclimate parameters in all other sensors was disregarded; however, these three sensors show microclimate variations changes throughout the greenhouse in over a period of 24 h at an average recorded change time every minute. Contour maps of the average hourly horizontal distribution of the optimality degrees were plotted for the minimum and maximum VPD per day to facilitate investigation and analysis.

### 3.1. Variation in Raw Microclimate Data 

**Figure 7:**
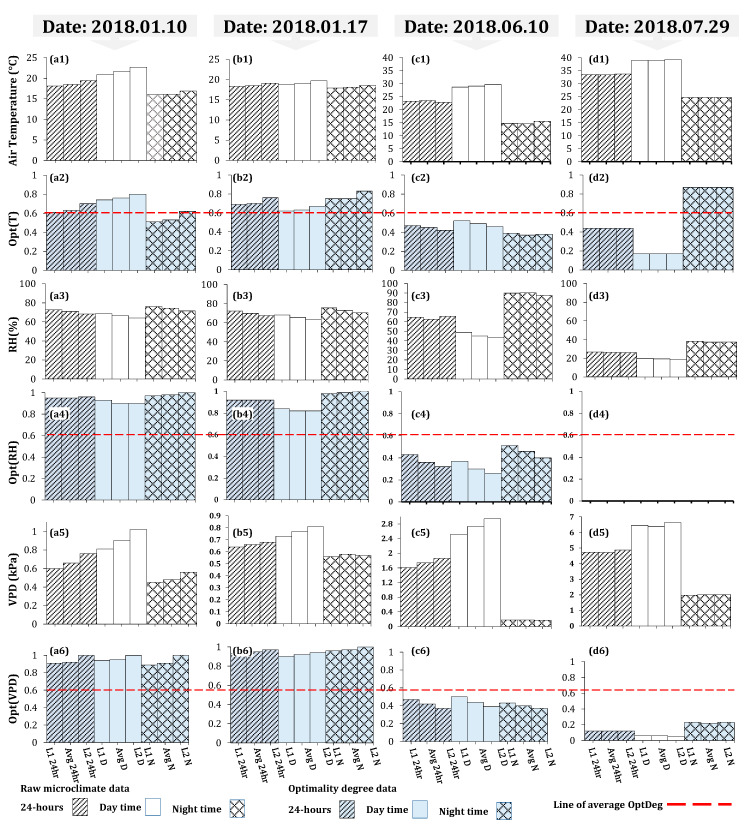
Average values optimality degrees of temperature, relative humidity and VPD. (**a1**) air temperature; (**a2**) OptDeg(T); (**a3**) relative humidity; (**a4**) OptDeg(RH); (**a5**) vapor pressure deficit and (**a6**) OptDeg(VPD) at 10 January 2018. (**b1**) air temperature; (**b2**) OptDeg(T); (**b3**) relative humidity; (**b4**) OptDeg(RH); (**b5**) vapor pressure deficit and (**b6**) OptDeg(VPD) at 17 January 2018. (**c1**) air temperature; (**c2**) OptDeg(T); (**c3**) relative humidity; (**c4**) OptDeg(RH); (**c5**) vapor pressure deficit and (**c6**) OptDeg(VPD) at 10 June 2018. (**d1**) air temperature; (**d2**) OptDeg(T); (**d3**) relative humidity; (**d4**) OptDeg(RH); (**d5**) vapor pressure deficit and (**d6**) OptDeg(VPD) at 29 July 2018. . L1 and L2 are 1.4 and 2.2 m above the ground in 10 January 2018, 17 January 2018 and 10 June 2018; L1 and L2 are 0.6 and 2.2 m height in 29 July 2018; D, N and 24 hr are day, night, day and night, respectively; Avg denote average. The dashed line is OptDeg average in total measurements.

**Figure 8:**
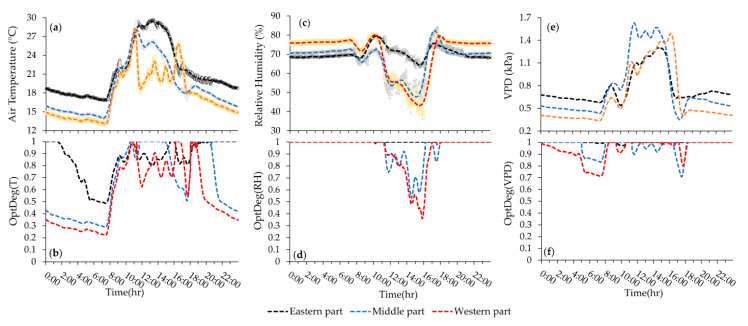
Hourly average variations of microclimate parameters and OptDeg at 10 January 2018. (**a**) air temperature; (**b**) OptDeg(T); (**c**) relative humidity; (**d**) OptDeg(RH); (**e**) vapor pressure deficit and (**f**) OptDeg(VPD).

**Figure 9:**
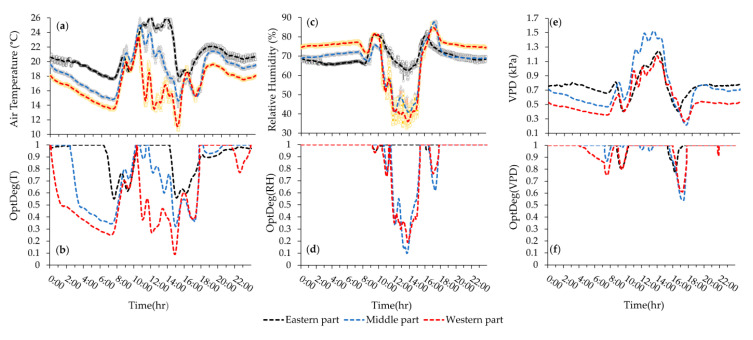
Hourly average variations of microclimate parameters and OptDeg at 17 January 2018. (**a**) air temperature; (**b**) OptDeg(T); (**c**) relative humidity; (**d**) OptDeg(RH); (**e**) vapor pressure deficit and (**f**) OptDeg(VPD).

**Figure 10:**
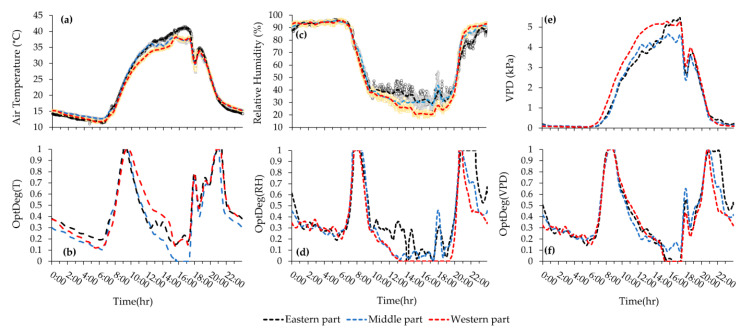
Hourly average variations of microclimate parameters and OptDeg at 10 June 2018. (**a**) air temperature; (**b**) OptDeg(T); (**c**) relative humidity; (**d**) OptDeg(RH); (**e**) vapor pressure deficit and (**f**) OptDeg(VPD).

**Figure 11:**
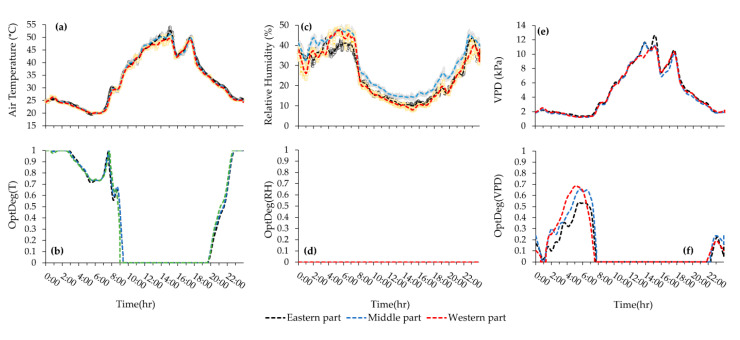
Hourly average variations of microclimate parameters and OptDeg at 29 July 2018. (**a**) air temperature; (**b**) OptDeg(T); (**c**) relative humidity; (**d**) OptDeg(RH); (**e**) vapor pressure deficit and (**f**) OptDeg(VPD).

In the first measurement, the western and eastern side wall vents were opened at 0.3 m (12.6 m^2^) and 0.4 m (20.4 m^2^) and in the second measurement they were opened at 0.4 m and 0.3 m respectively. Descriptive statistics of raw data and corresponding optimality degrees were generated for each day as reported in Figure 7a–c and Table 4. Plots of hourly averaged T, RH, VPD and corresponding optimality degrees are respectively shown in Figure 8, Figure 9, Figure 10 and Figure 11. The pattern of temperature changes in the two measurements of the cold season was the same (Figure 8a and Figure 9a). The hourly temperature changes measured by sensor number 26 were affected by inlet air temperature fluctuations due to being close to the air inlet ventilation vent. Near the eastern side of the greenhouse, the measured hourly temperature changes in sensors 16 and 6 were less affected by the fluctuations in the temperature of the air entering the greenhouse (Figure 8a and Figure 9a). An examination of the climatic conditions showed that the average temperature over 24-h, during the daytime and night time was not in the optimal range (Table 4 and Figure 7). The average relative humidity over 24-h in the two winter measurements showed similar values at approximately 70% (Table 4, Figure 7a3,b3), which were in the optimal range for the tomato crop (60% to 80%). Because of the decrease in temperature and the closing of the ventilation vents during the night, the average relative humidity at night was about 7% higher than the daily relative humidity (Table 4, Figure 7a3,b3). These results showed that the average relative humidity during the day and during the night was not within the optimal range for the tomato crop (Figure 7c3). Alongside the temperature reduction in the greenhouse, the humidity increased during the night. At night, the lowest VPD average was observed in all measurements due to the low temperature (14.7 °C) and high humidity (90.3%). If the heaters were on, the temperature remained within a favorable range and the humidity decreased. Even with the exhaust fans switched on at night, it was possible for the warmer air (17.0 °C) with less external humidity (61.7%) (Table 4) enter the greenhouse and reduce the extra humidity. For the early growth period, the lower optimal and upper optimal value is 24.0 and 26.1 °C and the lower and upper failure value is 9 °C and 35 °C [29]; hence, the temperature (24.7 °C) during the nighttime was within the favorable range (Table 4 and Figure 7d1).

Over 24-h, the hourly average temperature fluctuated from 14.3 °C at 6:00 to 24.8 °C at 11:00. In the first measurement, sensor 26 showed an increase in the temperature around 16:00. This increase in temperature could be seen in all sensors in the western part of the greenhouse. This was due to the earlier operation of a hot air heater in this area. Four hot air heaters were switched on at about 16:30 to 8:00 heating the greenhouse when it appeared to be a temperature loss during the night (Figure 8). The reason for more homogeneity at night was the closure of the vents and the function of the hot air heater (Figure 8a,b). The temperature distribution in the greenhouse at night time showed that a large area (more than 50%) of the greenhouse was below 17 °C (Figure 8a). At 20:00 this microclimate variance was limited to a small area and gradually spread until 8:00. The mean temperature was 1.8 and 0.9 °C at an elevation of 2.2 m more than at an elevation of 1.4 m high during the day and night (Figure 7a1). This indicates that at night and at an elevation of 1.4 m, the surface area of the greenhouse, which experienced unfavorable temperature conditions, was higher than that at an elevation of 2.2 m.

In the second measurement, due to the surrounding ambient air temperature of the greenhouse (11.2 °C) at 15:00, the temperatures recorded by these 3 sensors were at their lowest value. When the vents closed, the temperature increased but with the approach of sunset, the temperature fell again, which led to the turning on of the heaters at around 16:30. The 24-h average temperature recorded on this date was the same as the pervious measurement (Table 4). Although the 24-h average temperature was slightly over that of the minimum proper, the daytime temperature was less than that of the appropriate range of temperature. The temporal range of this reduction is lower than the previous measurement indicating that a higher temperature exists during the nighttime. The standard deviation (S.D. = 2.3 °C) shows a higher temperature homogeneity over the 24-h period as compared to the previous measurement (S.D. = 3.5 °C), due to the difference in day and night temperature (0.9 °C) as compared to that of the pervious measurement (5.5 °C). The temperature mean variation showed that in most hours of the day, the temperature average was greater than the accepted minimum while during the day, the temperature was over 19 °C (daytime minimum) for 5 h (Figure 9a). The average temperature at night (18.1 °C) was in the optimal range but during some hours of the night (Figure 9a), the greater area of the greenhouse (more than 50%) was at a less appropriate level. At 6:00–8:00 most of the greenhouse area experienced a lower favorable temperature (Figure 9a). It should be noted that the pattern in the variations of microclimate parameters and OptDegs observed in Figure 9 (date: 10 January 2018) is also similar to the one observed in Figure 8 (date: 10 January 2018). The trend of increasing temperature and relative humidity from west to east of the greenhouse was observed, especially during the day (Figure 8a,c and Figure 9a,c). During the daytime at 14:00 when the side wall vents were open, the hourly temperature and relative humidity in the greenhouse changed from 16.5 °C to 27.8 °C and 36.7% to 65.7% in the first measurement and 12.9 °C to 21.7 °C and 36.1% to 66.6% in the second measurement from the west-vent to the east-vent.

Hourly average variation of microclimate data and their associated OptDeg values for the experiment of 10th June 2018 (third measurement) is shown in Figure 10a–f. Similarly, the plots of data for the fourth measurement (date 29th July 2018) is shown in Figure 11a–f. A comparison between the third and fourth measurement shows that the pattern in temporal variation of air temperature in both experiment during the warm season has be the same (Figure 10a and Figure 11a). Another observation is that because of the large area affected by mechanical ventilation, the temperatures measured by sensors 6, 16 and 26 were very close to each other. In the fourth measurement, due to the presence of seedlings, the greenhouse area was nearly empty and as a result, hourly changes in temperature corresponded with each other. However, in these two measurements, during the peak hours of radiation, the temperature measured by sensor 6 was higher than that of the other two sensors. The western, eastern and end (north) vents were open at elevations 0.26 m (10.9 m^2^), 0.12 m (6.1 m^2^) and 1.17 m (103.5 m^2^) in the third measurement. Out to the working fans, the side wall vents were opened less so that the air flow entered the greenhouse from the end vent and, passed over the crop rows, where the exhaust fans extracted the high heat and humidity. The average 24-h temperature (23.5 °C) was suitable but the daytime average temperature (29.1 °C) over the favorable range and the nighttime average temperature (14.7 °C) might have prevented crop growth (Table 4 and Figure 7c1). On the other hand, the standard deviation of the 24-h temperature (S. D. = 9.6) showed a severe variation of temperature over 24-h in as such that the temperature average during the day was 14.4 °C higher than that at night (Table 4, Figure 7c1 and Figure 10a). During most hours of the day, the temperature was higher than 25 °C and at night less than 17 °C, which did not provide a suitable microclimate condition for tomato growth (Figure 10a). A review of the horizontal distribution of temperature in the greenhouse showed that at 5:00 (the coolest hour), the average temperature is less than 15 °C and these conditions began from 22:00 in some parts and extended to more than 80% of the greenhouse area by 7:00. From 9:00 to 19:00 the temperature was over 25 °C and from 12:00 to 17:00 the temperature increased to over 34 °C. Despite these conditions, measurement was possible and the S.D. = 1.07 of the horizontal distribution of temperature at every hour showed more homogeneity, which is consistent with the results of Kittas et al. [33]. 

### 3.2. Vapour Pressure Deficit Analysis

The largest hourly average VPD was 5.61 kPa, observed at 16:00. In the fourth measurement, the average 24-h temperature (33.4 °C) was greater than the favorable range for tomato cultivation in the early growth period. The average temperature during the daytime (38.8 °C) was greater than the upper marginal temperature. During a few hours at night, the temperature was between 19 °C and 20 °C (from 5:00 to 7:00) whereas the temperature was over 35 °C from 9:00 to 19:00 (Figure 11a). The hourly average temperature range varied from 19.6 °C to 51.2 °C (Table 4) over 24-h while the maximum temperature should not exceed 35 °C. The examination of the temperature spatial distribution during the coolest and warmest hours showed that the temperature was 19.1 °C to 20.6 °C during the coolest hours and 45 °C to 51.5 °C during the warmest hours (Figure 11a), thus creating a high heterogeneity (S.D. = 10.0). The average temperature outside the greenhouse was 36.8 °C (19.8–51.2 °C range) during the day and during the night it was 27.2 °C (19.6–31.7 °C range). The average night temperature inside the greenhouse was 24.7 °C which was less than the ambient temperature (Table 4), moreover, the average wind velocity at night was 0.68 ms^−1^, which in itself was considered to be a low air flow (wind velocity from 0.3 to 1.5 ms^−1^). Since the sky was clear during this period, there seemed to be thermal inversion. The relative humidity at night inside the greenhouse and the surrounding environment was 37.3% and 23.0%, respectively (Table 4). Based on this measurement, the ambient temperature average during the day (36.8 °C) was greater than the permissible maximum value and the relative humidity (18.9%) was less than the permissible minimum. The forced ventilation system was not capable of reducing the temperature and increase humidity. Zabeltitz [34] reported that evaporation cooling is necessary if the ambient temperature is greater than 36°C while the humidity is less than 60–55%. The pattern of changes for the VPD in the two cold season measurements was the same (Figure 8e and Figure 9e). In the cold season measurements, the average VPD during the daytime and during the nighttime was within the favorable range (Table 4, Figure 7a5,b5). The average VPD during the day was greater than during the night and greater at an elevation of 2.2 m than at an elevation of 1.4 m (Figure 7a5,b5). The VPD distribution during the different hours of the day showed that while the VPD average was within the favorable range at night in the western section of the greenhouse, the VPD was less than 0.41 (Figure 8e and Figure 9e).

After the vents were closed at around 16:00, a decrease in the amount of VPD, was observed which was compensated after the hot air heaters were turned on. During the day time, the hourly VPD was less than 1.43 kPa. At night, the amount of VPD increased from the west to the east of the greenhouse. During the day, the highest amount of VPD was observed in the center of the greenhouse, while in the eastern and western parts of the greenhouse, the amount of VPD was almost the same (Figure 8e and Figure 9e). As can be seen, while the air temperature in the center of the greenhouse was higher than in the western part (Figure 8a and Figure 9a), the amount of moisture in these two parts showed no difference (Figure 8c and Figure 9c); thus in terms of humidity, the rate was almost equal to that of the higher temperature, yet VPD in the center of the greenhouse was higher. In the eastern part of the greenhouse, concurrent with the increase in temperature, there could be seen an increase in humidity compared to the western part and the center of the greenhouse, which reduced the VPD in this part. Between the opening of the side wall vents at 8:30 until about noon when the air temperature inside the greenhouse started increasing, the intrusion of cold weather from the western ventilation vent, caused a decrease in the humidity weather maintenance capacity by reducing the temperature [35]; thus, the value of VPD was lower in the eastern part of the greenhouse (Figure 8e and Figure 9e). A review of the VPD horizontal distribution at 6:00 showed that the western section of the greenhouse had lower VPD values than the eastern part (Figure 9e). The examination of the VPD distribution at 14:00 showed that the greenhouse was in favorable conditions. Although VPD distribution was the same on these two dates, the value of VPD on 17 January 2018 (1.01 kPa) was less than that on 10 January 2018 (1.33 kPa) due to the conditions of the air intrusion. At 14:00, the temperature during the first and second measurements was 16.5 °C and 12.9 °C and the relative humidity was 36.7 and 38.1% in the western vent. The greater temperature at relatively the same humidity brought about greater dryness. In the third measurement, the average VPD during the day and during the night was 2.73 kPa (dry stress) and 0.18 kPa (high humidity), respectively (Table 4). This great difference between the VPD at night and during the day brought about intense S.D. (1.70) (Table 4). The average VPD over 24-h (1.74 kPa) was higher than the maximum allowed value. The VPD declined to less than 0.2 kPa from 22:00 continuing to 6:00 (Figure 10e). The lowest hourly VPD average was 0.06 kPa at 5:00 with the greatest relative humidity (95.5%) and lowest hourly average temperature (12.3 °C) recorded at that time. In June, the heaters were turned off and the hourly average temperature in the greenhouse at night was 14.7 °C, ranging between 12.7–17.7 °C). The hourly average night temperature outside was 17.0 °C (13.0–19.6 °C range). The reason was attributed to thermal inversion which occurred on nights with a clear sky and no convection and the radiation loss from the greenhouse to the sky is greater than the amount of its intrusion [36]. The natural ventilation in this situation was not significant due to the low air circulation (outside wind velocity was below 0.3 ms^−1^) and the thermal inversion (cooler and heavier air inside the greenhouse). In the fourth measurement, the VPD average at all conditions was greater than the allowed value (Figure 7d5 and Figure 11e). At the early growth period, the minimum and maximum of favorable ranges are 0.29 and 0.845 kPa and the value over which VPD is destructive is 2.248 kPa [29]. The thermal inversion made a lower VPD at night, although the VPD was still greater than the permissible value and there was no control over it. Over all hours, the hour average VPD ≥ 1.27 kPa was greater than the allowed value (Figure 11e).

### 3.3. Variations in Optimality Degrees of Microclimate Parameters

Analyzing raw data to determine the proximity of greenhouse climatic conditions to favorable conditions is difficult and time-consuming for greenhouse operators. Optimality degrees provide the user with a powerful tool to quickly identify greenhouse conditions numerically or graphically, which allows the user to make quick decisions about the climatic conditions of the greenhouse. Optimality degrees can also help researchers to analyze microclimate data more accurately and quickly in investigative studies.

The optimality degrees of the 24-h temperature during the two cold season measurements at a temperature of 18.5 was 0.63 and 0.70 (Figure 7a2,b2), respectively, which showed that in the second measurement, despite the equality of the average temperature, the temperature conditions were somewhat more optimal. Day and night optimality degrees in the first and second measurements were (OptDeg(T)_N_ = 0.53, OptDeg(T)_D_ = 0.76) and (OptDeg(T)_N_ = 0.75, OptDeg(T)_D_ = 0.63). The standard deviation of OptDeg(T)_24_ in these two measurements was 0.29 and 0.22, respectively, which indicates more non-uniformity of the temperature optimality degrees in the first measurement than in the second measurement. Examination of OptDeg(T) graphs for the cold season in terms of time changes showed that the optimality degrees were highly variable, indicating insufficient control of the greenhouse temperature. The lowest value of OptDeg(T) occurs at two periods, one close to 7:30 and the other around 17:30. Sharp fluctuations between these two periods can also be seen, especially in the second measurement (Figure 8b and Figure 9b). Examination of Figure 8b and Figure 9b showed that in the first and second measurements, when moving from the west to the east of the greenhouse, OptDeg(T) increased. In addition, more than 80% of the greenhouse at night had a temperature optimality degree of less than 0.5. During the day, OptDeg(T)_D_ = 0.6 was obtained approximately for the total area of the greenhouse and for more than 60% of the greenhouse area OptDeg(T) was observed to be > 0.8. In the second measurement, about 80% of the greenhouse area had an OptDeg(T) < 0.8. Most of the day and night, in the eastern part of the greenhouse OptDeg(T) > 0.9 was recorded. The optimum temperature for a 24-h period, during the day and night at a 2.2 m altitude showed more appropriate climatic conditions and uniformity than at an elevation of 1.4 m (Figure 7a2,b2). The phenomena can be attributed to the hot air blown in by the heating system. It is of note that the hot air furnace outlet was at an elevation of about 2.2 m (Figure 1d). 

In the third measurement, the average 24-h of OptDeg(T) was 0.45 (Figure 7c2). The average 24-h temperature was 23.5 °C at nights as opposed to the minimum optimum daily temperature (24 °C). OptDeg(T) at 1.4 m (0.52) was higher than at 2.2 m (0.46) (Figure 7c2). This can be attributed to the fact that better ventilation existed due to the installation height of the fans. Fans with a diameter of 1.4 m were installed at a height of 1 m and the fan center was approximately 1.7 m high. At night, the OptDeg(T) at two elevations of 1.4 (0.39) and 2.4 m (0.38) was approximately the same (Figure 7c2). The reason was the calm of the air and the phenomenon of thermal inversion. OptDeg(T) in most of the greenhouse and during the day and at night was less than 0.4 (Figure 10b). In general, the OptDeg(T) values were close to each other in the greenhouse during most hours (Figure 10b). In the fourth measurement, the average 24-h temperature was 33.4 °C (Table 4) and the average 24-h OptDeg(T) = 0.44 (Figure 7d2). While in the third measurement the difference between OptDeg(T)_D_ and OptDeg(T)_N_ was 0.12, in the fourth measurement this value was 0.70. The average night temperature was 24.7 °C which was in the optimal range for the tomato crop with a S.D. = 3.0. It was shown that at night the temperature was around an optimal range with ± 3.0 °C and the temperature optimality degrees for the night (87%) was higher on this date causing the average OptDeg(T) to rise. The average daily temperature was 38.8 °C (OptDeg(T) = 0.17) with a 9.0 standard deviation, which indicates the temperature, ± 9.0 °C changes around the average temperature. At night, almost the entirety of the greenhouse, OptDeg(T) was higher than 0.7 and in some hours its value reached 1.0. During the day, OptDeg(T) was zero throughout the greenhouse (Figure 11b). The amount of OptDeg(T)_D_ or OptDeg(T)_N_ varied depending on the climatic conditions of the greenhouse and was not necessarily considered as a trend which in itself indicates that OptDeg(T) was consistently higher during the day or night. The OptDeg(T) chart in the warm season and the third measurement showed that in general, the trend of changes was similar to the winter measurements, in as such that there is a decrease in OptDeg(T) at night and an increase during the day. The lowest OptDeg(T) was also observed at 6:30 in the morning and around 17:00 (Figure 10b and Figure 11b). The optimality degrees of relative humidity in the two measurements of the cold season showed that the relative humidity was at an optimal level. The OptDeg(RH) chart in winter measurements showed that the relative humidity was very good at night and was practically 1.0. The OptDeg(RH) pattern changes with time were similar in both measurements. OptDeg(RH) in the greenhouse reduced during the day when opening the side vents and reduced the humidity inside the greenhouse (Figure 8d and Figure 9d). OptDeg(RH) was approximately equal in day and night at the two measuring elevations (Figure 7(a4,b4)). In the first and second measurements over all hours of the day and night in the eastern part of the greenhouse, humidity conditions were optimal, that is OptDeg(RH) = 1.0 (Figure 8d and Figure 9d). The western and middle parts of the greenhouse also had an OptDeg(RH) = 1.0 at night but the OptDeg(RH) decreased during the day and reached to near 0.1 for a short time. In the first measurement, an OptDeg(RH) of less than 0.5 was observed at below 10% within the greenhouse area from 14:00 to 16:00. In the second measurement, from 12:00 to 15:00, at more than half of the greenhouse area experienced the OptDeg(T) < 0.5. In the third measurement, OptDeg(RH)_D_ and OptDeg(RH)_N_ were 0.30 and 0.46, respectively (Figure 7c4). OptDeg(RH) was low during the day due to scarcity and at night due to high humidity. During most hours of the day and night, within the greenhouse OptDeg(RH) < 0.3 (Figure 10d). From 14:00 to about 19:00 the entire surface of the greenhouse OptDeg(RH) was less than 0.1 and in the western part it was zero. In general, the OptDeg(RH) values were highly similar during most hours (Figure 10d). The diagram of OptDeg(RH) change with time shows that only during limited hours in the morning or evening when the relative humidity has decreased or increased with a large slope due to the turning off and on of the exhaust fans, did OptDeg(RH) increased and then decreased rapidly. In the fourth measurement, the OptDeg(RH) rating was zero, meaning that the amount of moisture was never appropriate for the early stages of tomato growth. 

**Figure 12:**
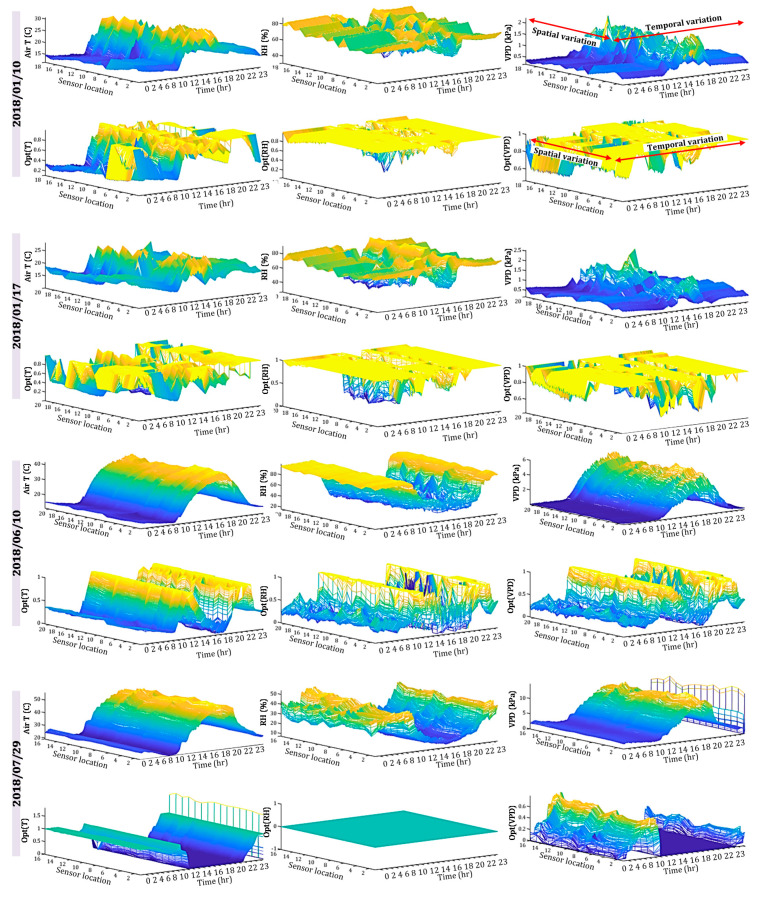
Temporal and spatial changes of temperature, relative humidity and vapor pressure deficit (VPD) and their corresponding optimality degrees in the measured points inside the greenhouse.

A more comprehensive representation of raw data and OptDeg data is provided by means of three-dimensional plots in Figure 12 that show the temporal and spatial changes of temperature, relative humidity and VPD and their corresponding OptDeg in all measured points inside the greenhouse. It should be noted that such an analyzing of raw data by local greenhouse growers and managers would have required extensive knowledge of the interaction between microclimate parameters and the plants, beside the regular time taking data processing procedure. 

These results clearly show that by deploying the wireless sensors that fuse their collected data into the AAF software for determining OptDeg, growers can have immediate access to processed data that translate each and every measurement of microclimate parameter into a number between 0 and 1, representing how close the greenhouse condition has been to optimal growth condition under that specific growth stage. The three-dimensional OptDeg plots provide shown in Figure 12 can be used to determine the spatial and temporal variations in the OptDeg of each specific parameter during the growth season. This helps managers to easily compare the performance of the heating and cooling systems in different cultivation days. According to Figure 12, the greenhouse under study has had a better performance in winter compared to the spring and summer season.

### 3.4. Variations in Optimality Degrees of VPD

Optimality degrees over a 24-h period and during the day and night measurements of VPD in the two measurements of the cold season, was between 0.91 and 1.0 with a standard deviation range of 0.06 to 0.11. These values showed that the VPD was in the optimal range. VPD optimality degrees had the same pattern in winter measurements during the day and night and the value was greater than 0.9 in most hours. This pattern was affected by the opening and closing of the vents and sunlight during the day (Figure 8f and Figure 9f). In the first measurement, the optimality degrees over a 24-h period and during the day and night measurements of VPD at an elevation of 2.2 m had a value of 1.0. The same trend was seen in the second measurement, in which the OptDeg(VPD) increased in the first and second measurements from the west to the east of the greenhouse and excepted for limited hours in the morning or at sunset, the value of the measurements throughout the greenhouse exceeded 0.9 (Figure 8f and Figure 9f). 

In the third measurement, the mean 24-h, day and night measurements of OptDeg(VPD), was 0.42, 0.44 and 0.40, respectively (Figure 7c6). OptDeg(VPD) was almost the same during the day and night but during the day due to high VPD, OptDeg(VPD) was reduced and the crop was subjected to drought stress but at night OptDeg(VPD) was lowered due to high humidity and conditions were ready for the damage of pathogens to the crop. The OptDeg(VPD) value changed during limited hours of the day and night and was influenced by the turning on and off of the fans. During the transition, temperature and humidity from one condition to another condition was optimum for a while but this situation was not sustainable (Figure 10f). From midnight to about 7:00 and from 12:30 to 17:30, the optimality degrees of VPD fell below 0.3 and in a few hours, the OptDeg(VPD) in the western and eastern parts of the greenhouse reached zero (Figure 10f). For limited hours in the 24-h period, the OptDeg(VPD) value increased to above 0.5. In the fourth measurement, the mean 24-h, day and night measurements of OptDeg(VPD), are 0.12, 0.06 and 0.22, respectively, indicating the unfavorable conditions of VPD for the crop on this date (Figure 7d6). The value of OptDeg(VPD) on the day mentioned was practically close to zero. OptDeg(VPD) at night was the same (0.23) at both elevations. At night, the microclimate improved with a decreasing in temperature and increasing humidity due to the thermal inversion phenomenon. Even during a limited time of the night, the OptDeg(VPD) through the entire greenhouse area was between 0.5 and 0.7. OptDeg(VPD) decreased during these hours from west to east of the greenhouse (Figure 11f). 

### 3.5. Horizontal Distribution of Microclimate and Optimality Degrees

**Figure 13:**
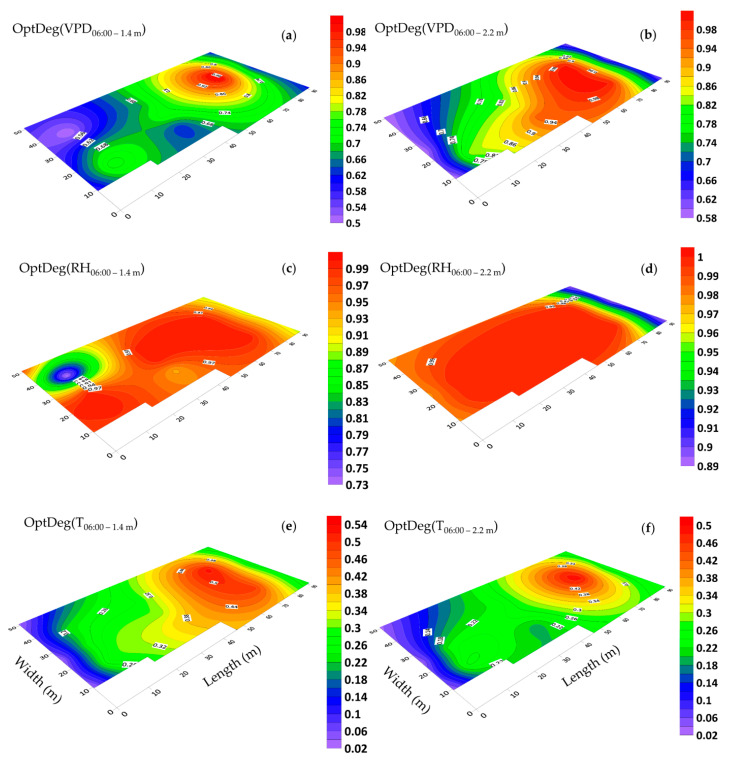
Optimality degrees **range and distribution **on **a horizontal plane** at 06:00 in 1.4 m and 2.2 m elevations on 10 January 2018. OptDeg(VPD) at 1.4 m (**a**) and 2.2 m (**b**); OptDeg(RH) at 1.4 m (**c**) and 2.2 m (**d**); and, OptDeg(T) at 1.4 m (**e**) and 2.2 m (**f**).

**Figure 14:**
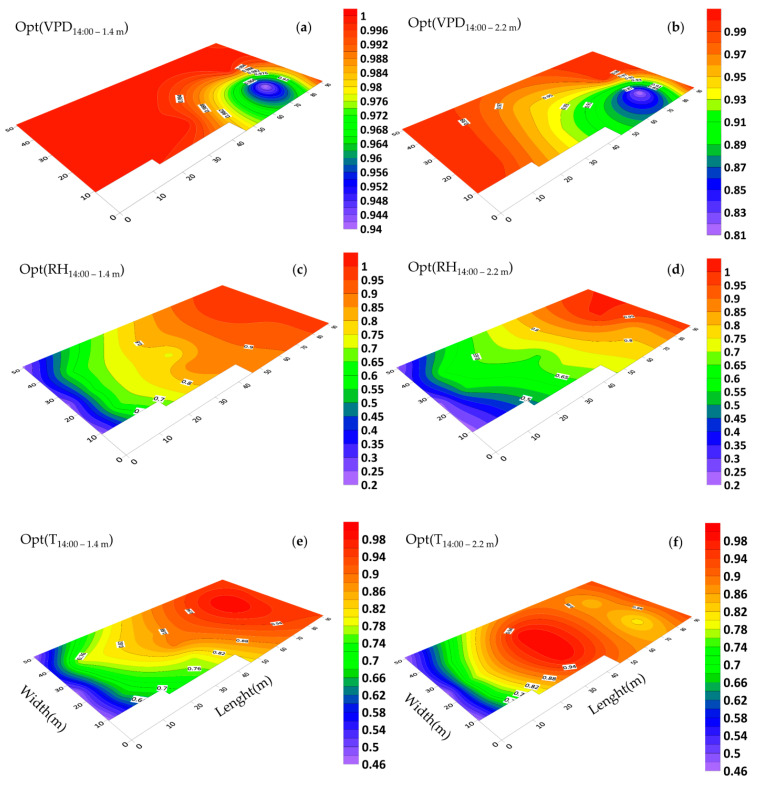
Optimality degrees **range and distribution** on **a horizontal plane** at 14:00 in 1.4 m and 2.2 m elevations on 10 January 2018. OptDeg(VPD) at 1.4 m (**a**) and 2.2 m (**b**); OptDeg(RH) at 1.4 m (**c**) and 2.2 m (**d**); and, OptDeg(T) at 1.4 m (**e**) and 2.2 m (**f**). Scales display the range of optimality degrees variation.

**Figure 15:**
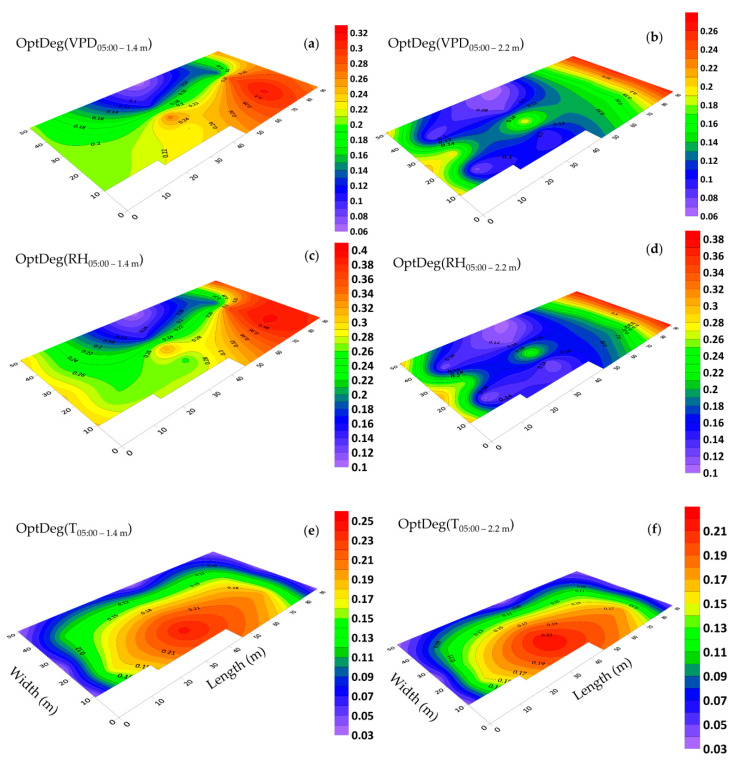
Optimality degrees **range and distribution** on **a horizontal plane** at 05:00 in 1.4 m and 2.2 m elevations on 10 June 2018. OptDeg(VPD) at 1.4 m (**a**) and 2.2 m (**b**); OptDeg(RH) at 1.4 m (**c**) and 2.2 m (**d**); and, OptDeg(T) at 1.4 m (**e**) and 2.2 m (**f**).

**Figure 16:**
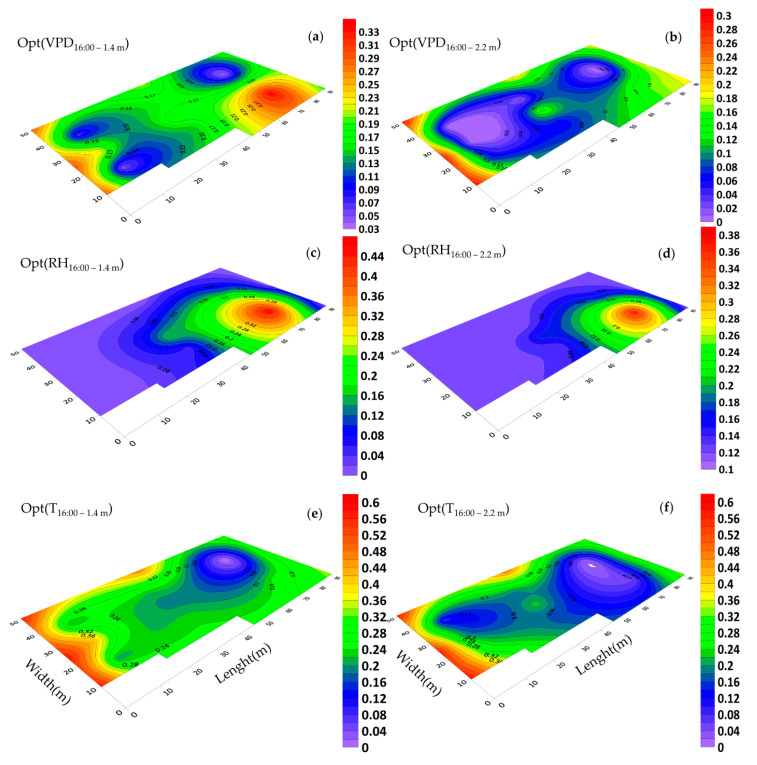
Optimality degrees **range and distribution** on **a horizontal plane** at 16:00 in 1.4 m and 2.2 m elevations on 10 June 2018. OptDeg(VPD) at 1.4 m (**a**) and 2.2 m (**b**); OptDeg(RH) at 1.4 m (**c**) and 2.2 m (**d**); and, OptDeg(T) at 1.4 m (**e**) and 2.2 m (**f**).

The horizontal distribution of the optimality degrees of microclimate parameters over two hours of the day and night, when the VPD value was within the minimum and maximum range, has been given in Figure 13, Figure 14, Figure 15 and Figure 16. The average hourly results were used to draw the graphs. For example, the mean measurement at 07:00 is the averaged data from 06:01 to 07:00, which is practically 3600 data per sensor. The figures were drawn on two dates 10 January 2018 (06:00 and 14:00) and 10 June 2018 (05:00 and 16:00) and at two elevations of 1.4 and 2.2 m. In the previous sections, by examining the data collected by the three sensors, the general trend of spatial changes of microclimate parameters and the optimality degrees were defined; however, the current section will look at the spatial distribution of optimality degree changes in the greenhouse. In the first measurement, the contour map of the OptDeg(T) at 06:00 showed that the general pattern of OptDeg(T) at elevations 1.4 and 2.2 m was the same (Figure 13e,f). Since the ventilation vents were closed at this time, the OptDeg(T) almost matched the shape of the greenhouse itself (Figure 2). At an elevation of 1.4 m, this juxtaposition was more pronounced. It was observed that the OptDeg(T) increased with a gentle slope from the west to the east of the greenhouse. Because of the larger special expanse in the eastern part of the greenhouse and the distance of the center of this section from the greenhouse walls (ambient air temperature −4.9 °C), temperature stability and maintaining the heat content was better and compared to other areas of the greenhouse the temperature optimality degree improved. However, at this time, the optimum temperature in the greenhouse was less than 0.5, which was indicative of a critical condition.

The OptDeg(RH) for both elevations in the greenhouse showed favorable conditions (Figure 13c,d). At an elevation of 1.4 m in the west of the greenhouse was a spot with a lower OptDeg(RH). In the northwestern part of the greenhouse, at an elevation of 1.4 and 2.2 m, the average hourly relative humidity was 85.5 and 79.6 percent, respectively. While at a height of 2.2 m the humidity condition of the greenhouse was in the optimal range, at the height of 1.4 m with OptDeg(RH) = 0.73 was outside this range (Figure 13c,d). Just as VPD is a function of temperature and relative humidity, the OptDeg(VPD) is also affected by the optimality degree of temperature and relative humidity. At both elevations, the OptDeg(VPD) in the eastern part of the greenhouse was higher than that in the western part (Figure 13a,b). At an elevation of 2.2 m, a gradual increase was observed in the OptDeg(VPD) from the west to the east of the greenhouse (Figure 13b). However, at a height of 1.4 m in the western part of the greenhouse, two spots were observed, in which the difference of the OptDeg(VPD) between these two spots was approximately 0.2 (Figure 13a). In general, at 06:00 on 01/10/2018, an area with a low VPD optimality degree can be seen in the western part of the greenhouse (0.50 to 0.70), a transition zone in the middle of the greenhouse was also identified (0.7 to 0.8) and in the eastern part of the greenhouse an area with a high VPD optimality degree (0.8 to 0.1) was observed (Figure 13a,b).

At 14:00, when the ventilation vents in the western and eastern walls of the greenhouse were open, the optimality degree of the temperature and the relative humidity increased from the west (0.50) near the ventilation inlet vent to the east (1.00) (Figure 14c–f). The optimality degree of temperature, relative humidity and VPD at 1.4 m was higher than the elevation of 2.2 m (Figure 14a–f). At an elevation of 1.4 m, about 70% of the greenhouse area had OptDeg(VPD) equal to 1.0. At both elevations in the southeast of the greenhouse, there was a spot where the gradient of the OptDeg(VPD) was steep. It can be said that from the southeast to the northwest of the greenhouse, there was an increase in the OptDeg(VPD) (Figure 14a,b) and in line with that, was the increasing of the optimality degree of temperature and relative humidity in the opposite direction (Figure 14c–f).

During the warm season on 10 June 2018 the lowest and highest VPD values recorded were at 05:00 and 16:00 respectively thus these periods were selected for investigation. At 05:00, the sides and rear (north) vents of the greenhouse were open but the exhaust fans were off. The OptDeg(T) was approximately the same at the two elevations of 1.4 and 2.2 m and indicated the critical temperature conditions in the greenhouse (Figure 15e,f) but its pattern varies for the two elevations specified. The OptDeg(RH) showed an increasing trend from the west to the east of the greenhouse. A review of the data showed that in the middle of the greenhouse, the amount of relative humidity measured at a height of 2.2 m in the northern, central and southern parts was 0.6% (97.65% and 97.07%), 1.7% (95.21% and 93.57) and 2.7% (% 97.59 and 94.89), higher than at the elevation of 1.4 m, respectively. A similar minor difference in the southern section towards the middle of the greenhouse resulted into a 2-fold increase in the OptDeg(RH) at a height of 1.4 m as compared to the data observed at an elevation of 2.2 m (Figure 15c,e). However, in general, the OptDeg(RH) was very low, indicating that the greenhouse in terms of humidity was in a critical condition. 

At 16:00, the sides and back (north) walls, vents of the greenhouse were open and the exhaust fans were on. Temperature optimality degree distribution indicated critical temperature conditions in the greenhouse (Figure 16e,f).

In general, the contour map of the temperature optimality degree showed that the temperature condition at an elevation of 1.4 m had far more favorable conditions than that at an elevation of 2.2 m, especially in the western part of the greenhouse. As previously indicated, the underlying reason for such a variation was better ventilation at an elevation of 1.4 m due to the installation height of the exhaust fans and the height of the end vent. In the western part of the greenhouse, due to the shorter length of the spans, there was better ventilation and as a result, the temperature optimality degree was better. In the western part and at a height of 1.4 m, the temperature in the northern, central and southern parts of the greenhouse was 37.2 °C, 34.5 °C and 37.4 °C, respectively and the temperature optimality degree was 0.24, 0.36 and 0.22. This means that with a slight variation in temperature, the temperature optimality degree can be increased to 0.1 (10%).

The OptDeg(RH) throughout most of the greenhouse area was less than 0.1. In the southeastern section of the greenhouse due to its extensive area, the OptDeg(RH) was higher than the other sections within the greenhouse. This was largely due to the low suction of the air by the exhaust fans and a high amount of vegetation and subsequent transpiration, which in itself resulted into the moisture accumulating in this part (Figure 16c,d). However, considering the relative humidity of the air around the greenhouse (25.5%), it can be said that the operation of the exhaust fans without the presence of a wet pad caused more dryness of the space inside the greenhouse. The OptDeg(VPD) had a pattern similar to the OptDeg(T) distribution pattern (Figure 16a,b,e,f) at 16:00, while at 05:00 it was mostly a function of the OptDeg(RH) (Figure 14a–d). Because OptDeg(T) at 05:00 and OptDeg(RH) at 16:00 were uniform, the pattern of the OptDeg(VPD) was affected by the non-uniformity of OptDeg(RH) at 05:00 and OptDeg(T) at 16:00. Throughout the major part of the greenhouse area, the OptDeg(VPD) was less than 0.2 at an elevation of 1.4 m and less than 0.1 at an elevation of 2.2 m.

## 4. Discussion

Mapping changes for the microclimate parameters of different parts of the greenhouse during the cold season showed that the pattern of their changes over different days had almost the same trend (Figure 8 and Figure 9). A comparison of the general trend for changes in microclimate parameters based on the data from three sensors (Figure 8 and Figure 9) and the contour map of microclimate parameters (Figure 13 and Figure 14) at two elevations in the greenhouse with 20 sensors showed that with a limited number of sensors the general trend of the change of microclimate parameters in the axis of the greenhouse can be determined, however, the details related to changes in the microclimate level of the greenhouse and the determining of the rate of homogeneity at various points at critical levels requires an appropriate number of sensors and suitable distribution. When the ventilation volume was higher or the height of the crop was low, not only the pattern but also the difference in the amount of the measured microclimate parameters along the greenhouse was minimal. As seen in Figure 8a,c, the greenhouse is divided into three zones in terms of temperature and humidity approximately coincident with their area. The greenhouse asymmetric shape and its division into three special areas of 2295, 1215 and 756 m^2^ (Figure 2) led to the uneven distribution of solar radiation and heat, as consistent with the studies carried by van Dam [23]. On the other hand, the inlet air from the west side wall vent (wind wise 230°) into the greenhouse, passed through the greenhouse, gradually becoming warmer and exited from the east side wall vent. Although the opening of the vent (33 m^2^) is 0.8% greenhouse area, was at a greater distance from the recommended portion of 25% [2], it caused the ventilation rate to slow down and resulted into the accumulation of heat in the eastern area of the greenhouse. The fact that there were three areas in the greenhouse with different transpiration rates resulted into a raise in values between 89% and 203% times in the old section more than the new sections; in other words, transpiration volume in the old section was far greater and when influenced by the wind flow, caused the humidity to move towards the east which, in turn, resulted into the accumulation of humidity in this area, due to the slow ventilation rate. 

The result was more heterogeneous during the day than at night (Table 4), when there was no sunlight, the vents were closed and the hot air heaters were on. The results obtained for measurements carried out during the cold season showed that the optimality degrees of microclimate parameters at an elevation of 2.2 m is greater than those obtained at a height of 1.4 m but in the third measurement in June, the obtained results were inverse. The reason for such a fact, as previously explained, was the elevation of the installation of greenhouse equipment such as hot air heaters and exhaust fans. In winter, the OptDeg(T) indicated that at night there was a need for more heating to optimize the temperature at the greenhouse. There was also a difference in the amount of heating required in different areas of the greenhouse. In the second measurement, the heating requirements of the eastern part of the greenhouse were chiefly provided. The OptDeg(T) during the day indicated that some hot air heaters were necessary to be turned on during the day to improve the greenhouse temperature. In the third measurement, the OptDeg(T) showed that the temperature needed to be improved due to the decrease and increase in the night and day temperatures, respectively. This deviation was more significant in the eastern part of the greenhouse, where the optimality degrees were nearly or equal to zero (Figure 16f). In moderate or cold climate conditions, however, the minimum OptDeg(T) values in the greenhouse were either due to high or low temperature values [26]. In fact, the minimum OptDeg(T) is an indication of the lowest tomato’s growth response to air temperature, which causes crop stress with a significant effect on yield and the development of fruit setting. These values are associated with critical hours in which maximum cooling is required [26,28]. Due to thermal inversion, the OptDeg(T) values in the fourth measurement at night were above 0.7 and in some hours were equal to 1.0. However, the OptDeg(T) during the day was zero, which indicated the need for a fundamental improvement of temperature conditions. This result is consistent with that of Sato et al. [37], who concluded that temperature exceeding 27 °C reduce tomato production [26].

Optimality degrees of the relative humidity in the two measurements of the cold season showed that the amount of greenhouse moisture was at an optimal level during the night (Figure 8d, Figure 9d and Figure 13d) but during the day in the middle and western part of the greenhouse, it had to be improved (Figure 8d, Figure 9d and Figure 14c). In the cold season, the OptDeg(RH) were higher than the temperature optimality degrees, which were consistent with the results of Shamshiri et al. [28] but in the warm season, it was vice versa. The OptDeg(RH) in the warm season generally indicate a critical situation in the greenhouse, which in combination with the OptDeg(T) conditions, made it necessary to use an evaporative cooling system to reduce the temperature and increase the humidity in the greenhouse. Therefore, it was concluded that the greenhouse under study require a method or combination of methods (i.e., shading, mechanical ventilating or even air conditioning) to control ambient temperature in these critical hours. VPD optimality degrees in the cold season showed that the combined effect of temperature and humidity, expressed as VPD, indicates better overall conditions than temperature and humidity separately. However, in the coldest or driest hours, the amount decreases. In the warm season, the OptDeg(VPD) is generally affected by the temperature and humidity and showed critical conditions [26] at the greenhouse.

In the two measurements of the warm season, despite different vegetation heights, the patterns of temperature, relative humidity and the VPD change were similar. The forced ventilation using outlet fans was deemed to be effective in creating such a similarity in this pattern. Relative humidity in the July measurements was lower than that in June but their pattern changes were the same over the 24-h period. It was observed that thermal inversion due to a clear sky, lack or low velocity of wind, caused reduction temperature and increased relative humidity inside the greenhouse as compared with the ambient environment. This condition showed the ineffectiveness of natural ventilation at night to produce favorable conditions. In the June measurement, it was possible the prevention of thermal inversion in the greenhouse by the utilization of equipment (heaters and exhaust fans) or polyethylene (IR-PE) [36], resulted in the improvement of the environment. But it was necessary to use evaporative cooling or a fog system in July, which was consistent with the study of Katsoulas et al. [32]. According to the solar radiation and the high temperature of the greenhouse ambient environment in the warm season, the utilization of exhaust fans (output aspirators) caused temperature and humidity uniformity just in the active hours of the day within the greenhouse and it did not help to improve environmental conditions. The investigation of climate conditions around and inside the specified greenhouse showed that ceiling windows and a cold evaporation system are necessary. The greenhouse grower refused to shade with a whitening coat of paint in the hot season in order to limit the increasing of heat by reducing the amount of radiation which reached the greenhouse. Hot weather also caused a disturbance in the pollination, abortion and fruit set of tomatoes and ultimately its yield reduction.

## 5. Conclusions

This paper employed IoT sensor fusion in combination with the optimality degree model for real-time dynamic assessment of greenhouse microclimate in commercial scale production of tomato. The method was based on the integration of wireless communication, distributed data analyzing and a web-based data monitoring dashboard that was used for data collection, processing and monitoring. The presented method was a proof-of-concept that showed the opportunity to use these new tools for a detailed investigation of the spatial and temporal variations in the air temperature, relative humidity and VPD inside greenhouse crop production. The implication is to provide growers with digital tools that can assist in knowledge-based decision making for minimizing energy cost and yield loss due to low fruit quality. Moreover, the IoT automation system and cloud data processing contribute as real-time online assessment tool to investigate effects of structure design, covering materials, cooling techniques and growing seasons on the optimality and comfortability of microclimate parameters and their correlation with yields. Results of this study showed that average optimality degrees of temperature, relative humidity and VPD in the cold season were 0.67, 0.94 and 0.93 respectively and in the warm season were 0.45, 0.0 and 0.27, respectively. In winter, the OptDeg(T) indicated that there was a need for more heating to optimize the temperature at the greenhouse. The OptDeg(T) in the warm season, in combination with the OptDeg(RH), generally showed a critical climatic condition in the greenhouse that indicated it was necessary to use an evaporative cooling system to reduce the temperature and increase the humidity in the greenhouse. Spatial variations in the optimality degree values in the vertical axis were found to be affected by the location and height of the greenhouse microclimate control equipment. The average optimality degrees of temperature, relative humidity and VPD at an altitude of 1.4 and 2.2 m in the cold season were 0.65, 0.935, 0.92 and 0.73, 0.94, 0.985 and in the warm season were 0.455, 0.215, 0.295 and 0.43, 0.16, 0.245, respectively. Analysis of the horizontal distribution with OptDeg model showed that even with the average OptDeg between 0.8 and 1, there were still regions and hours in the greenhouse that resulted microclimate with extremely low OptDeg values below 0.3, which were denoted as critical points and times that require attention by growers. Outcomes of this study implies that light condition at different growth stages, as well as shape of the greenhouse and locations of the heating and cooling system have significant impact on resulting a non-uniform microclimatic distribution. This study can be extended by combining the OptDeg model with existing computational fluid dynamics (CFD) model for simulating air flow and analyzing internal greenhouse microclimate.

## Figures and Tables

**Table 1:** Reference values of air temperature, relative humidity and vapor pressure deficit in different growth stages and light conditions for tomato [16,26,29,31]. G0: index of failure, G0.5: index of OptDeg = 0.5, G1: index of OptDeg = 1.0, min: lower border, max: higher border.

Growth Stage	Temperature		Relative Humidity		Vapor Pressure Deficit	
	Border	Value (°C)	Border	Value (%)	Border	Value (kPa)
Stage 1	*T*1*_G0min_*	9	*RH*1*_G0min_*	60	*VPD*1*_G0min_*	0.011
	*T*1*_G0max_*	35	*RH*1*_G1max_*	75	*VPD*1*_G0max_*	2.248
	*T*1*_G1min_*	24	*RH*1*_G1max_*	99	*VPD*1*_G1min_*	0.030
	*T*1*_G1max_*	26.1			*VPD*1*_G1max_*	0.845
Stage2	*T*2*_G0min_*	10	*RH*2*_G0min_*	40	*VPD*2*_G0min_*	0.012
	*T*2*_G0max_*	40	*RH*2*_G0max_*	99	*VPD*2*_G0max_*	4.422
	*T*2*_G0.5_*_(*night*)_	17	*RH*2*_G1min_*	70	*VPD*2*_G1min(sun)_*	0.596
	*T*2*_G1min(night)_*	18	*RH*2*_G1max_*	80	*VPD*2*_G1max_*_(*sun*)_	1.069
	*T*2*_G1max_*_(*night*)_	20			*VPD*2*_G1min_*_(*cloud*)_	0.528
	*T*2*_G1min_*_(*sun*)_	24			*VPD*2*_G1max_*_(*cloud*)_	0.895
	*T*2*_G1max_*_(*sun*)_	27			*VPD*2*_G1min_*_(*night*)_	0.412
	*T*2*_G1min_*_(*cloud*)_	22			*VPD*2*_G1max_*_(*night*)_	0.701
	*T*2*_G1max_*_(*cloud*)_	24				
Stage 3 to 5			*RH*3*_G0min_*	30	*VPD*3*_G0min_*	0.012
	Same as stage 2		*RH*3*_G0max_*	99	*VPD*3*_G0max_*	5.160
			*RH*3*_G1min_*	60	*VPD*3*_G1min(sun)_*	0.596
			*RH*3*_G1max_*	80	*VPD*3*_G1max_*_(*sun*)_	1.425
					*VPD*3*_G1min_*_(*cloud*)_	0.528
					*VPD*3*_G1max_*_(*cloud*)_	1.193
					*VPD*3*_G1min_*_(*night*)_	0.413
					*VPD*3*_G1max_*_(*night*)_	0.935

**Table 2:** Mathematical descriptions of the membership functions defining optimality degrees of air temperature, relative humidity [16,26,29]. G0: index of failure, G1: Growth stage1, G2: Growth stage2, G3: Growth stage3, A: All light conditions, N: Night, C: Cloud, S: Sun.

Membership Function	Range of Function
$OptDeg{\left(T\right)}_{G1A}=\{\begin{array}{c} 0\\ \frac{T-T1_{G0,min}}{T1_{G1,min}-T1_{G0,min}}\\ 1\\ \frac{-\left(T-T1_{G0,max}\right)}{T1_{G0,max}-T1_{G1,max}} \end{array}$	$T\langle T1_{G0,min\;}\;and\;\;T\rangle T1_{G0,max}$ $T1_{G0,min\;}\;\leq \;T<T1_{G0,min}$ $T1_{G1,min\;}\;\leq \;T\leq T1_{G1,max}$ $T1_{G1,max\;}<T\leq T1_{G0,max}$
$OptDeg{\left(T\right)}_{G2S}=\{\begin{array}{c} 0\\ \frac{T-T2_{G0,min}}{T2_{G1,\min\left(sun\right)}-T2_{G0,min}}\\ 1\\ \frac{-\left(T-T2_{G0,max}\right)}{T2_{G0,max}-T2_{G1,\max\left(sun\right)}} \end{array}$	$T\langle T2_{G0,min\;}\;and\;\;T\rangle T2_{G0,max}$ $T2_{G0,min\;}\;\leq \;T<T2_{G1,\min\left(sun\right)}$ $T1_{G1,\min\left(sun\right)\;}\;\leq \;T\leq T1_{G1,\max\left(sun\right)}$ $T1_{G1,max\;\left(sun\right)}<T\leq T1_{G0,max}$
$OptDeg{\left(T\right)}_{G2N}=\{\begin{array}{c} 0\\ \frac{0.5\left(T-T2_{G0,min}\right)}{T2_{G0.5,\left(night\right)}-T2_{G0,min}}\\ \frac{0.5\left(T-T2_{G0,min}\right)}{T2_{G0.5,\left(night\right)}-T2_{G0,min}}\\ 1\\ \frac{-\left(T-T2_{G0,max}\right)}{T2_{G0,max}-T2_{G1,\max\left(night\right)}} \end{array}$	$T\langle T2_{G0,min\;}\;and\;\;T\rangle T2_{G0,max}$ $T2_{G0,min\;}\;\leq \;T<T2_{G0.5,\left(night\right)}$ $T2_{G0.5,\left(night\right)\;}\leq \;T<T2_{G1,\min\left(night\right)}$ $T2_{G1,min\left(night\right)\;}\leq \;T\leq T2_{G1,\max\left(night\right)}$ $T2_{G1,max\left(night\right)\;}\leq \;T\leq T2_{G1,\text{max}}$
$OptDeg{\left(T\right)}_{G2S}=\{\begin{array}{c} 0\\ \frac{T-T2_{G0,min}}{T2_{G1,\min\left(cloud\right)}-T2_{G0,min}}\\ 1\\ \frac{-\left(T-T2_{G0,max}\right)}{T2_{G0,max}-T2_{G1,\max\left(cloud\right)}} \end{array}$	$T\langle T2_{G0,min\;}\;and\;\;T\rangle T2_{G0,max}$ $T2_{G0,min\;}\;\leq \;T<T2_{G1,\min\left(cloud\right)}$ $T1_{G1,\min\left(cloud\right)\;}\;\leq \;T\leq T1_{G1,\max\left(cloud\right)}$ $T1_{G1,max\;\left(cloud\right)}<T\leq T1_{G0,max}$
$OptDeg{\left(RH\right)}_{G1A}=\{\begin{array}{c} 0\\ \frac{RH-RH1_{G0}}{RH1_{G1,min}-RH1_{G0}}\\ 1 \end{array}$	$\;RH\langle RH1_{G0}$ $RH1_{G0\;}\;\leq \;RH\leq RH_{G1,\min}$ $RH\rangle RH1_{G1,min}$
$OptDeg{\left(RH\right)}_{G2A}=\{\begin{array}{c} 0\\ \frac{RH-RH2_{G0,min}}{RH2_{G1,min}-RH2_{G0,min}}\\ 1\\ \frac{-\left(RH-RH2_{G0,\max}\right)}{RH2_{G0,max}-RH2_{G1,max}} \end{array}$	$RH\langle RH2_{G0,min}\;and\;RH\rangle RH2_{G0,max}$ $RH2_{G0,min\;}\;\leq \;RH<RH_{G1,\min}$ $RH2_{G1,min\;}\;\leq \;RH\leq RH_{G1,\max}$ $RH2_{G1,max}<RH\leq RH_{G1,\max}$
$OptDeg{\left(RH\right)}_{G3A}=\{\begin{array}{c} 0\\ \frac{RH-RH3_{G0,min}}{RH3_{G1,min}-RH3_{G0,min}}\\ 1\\ \frac{-\left(RH-RH3_{G0,\max}\right)}{RH3_{G0,max}-RH3_{G1,max}} \end{array}$	$RH\langle RH2_{G0,min}\;and\;RH\rangle RH2_{G0,max}$ $RH2_{G0,min\;}\;\leq \;RH<RH_{G1,\min}$ $RH2_{G1,min\;}\;\leq \;RH\leq RH_{G1,\max}$ $RH2_{G1,max}<RH\leq RH_{G0,\max}$

**Table 3:** Mathematical descriptions of the membership functions defining optimality degrees of vapor pressure deficit [16,29,31]. G1: G0: index of failure, Growth stage1, G2: Growth stage2, G3: Growth stage3, A: All light conditions, N: Night, C: Cloud, S: Sun.

Membership Function	Range of Function
$OptDeg{\left(VPD\right)}_{G1A}=\{\begin{array}{c} 0\\ -1220vpd^{2}+103.6vpd-1.015\\ 1\\ -0.1046e^{0.5763vpd}+2.296e^{-0.799vpd} \end{array}$	$vpd\langle VPD1_{G0,min}\;and\;vpd\rangle VPD1_{G0,max}$ $VPD1_{G0,min\;}\;\leq \;vpd<VPD1_{G1,\min}$ $VPD1_{G1,min\;}\;\leq \;vpd\leq VPD1_{G1,\max}$ $VPD1_{G1,max}<vpd\leq VPD1_{G0,\max}$
$OptDeg{\left(VPD\right)}_{G2C}=\{\begin{array}{c} 0\\ -0.626e^{0.8509vpd}-0.6655e^{-4.974vpd}\\ 1\\ -0.0339e^{0.4326vpd}+1.711e^{-0.4562vpd} \end{array}$	$vpd\langle VPD2_{G0,min}\;and\;vpd\rangle VPD2_{G0,max}$ $VPD2_{G0,min\;}\;\leq \;vpd<VPD2_{G1,min\left(sun\right)}$ $VPD2_{G1,min\left(sun\right)\;}\;\leq \;vpd\leq VPD2_{G1,max\left(sun\right)}$ $VPD2_{G1,max\left(sun\right)}\;<\;vpd\leq VPD2_{G0,\text{max}}$
$OptDeg{\left(VPD\right)}_{G2C}=\{\begin{array}{c} 0\\ -0.6922e^{-4.889vpd}+0.6492e^{0.9212vpd}\\ 1\\ -0.01505e^{0.5616vpd}+1.591e^{-0.4981vpd} \end{array}$	$pd\langle VPD2_{G0,min}\;and\;vpd\rangle VPD2_{G0,max}$ $VPD2_{G0,min\;}\;\leq \;vpd<VPD2_{G1,min\left(cloud\right)}$ $VPD2_{G1,\text{min}\left(cloud\right)\;}\;\leq \;vpd\leq VPD2_{G1,max\left(cloud\right)}$ $VPD2_{G1,max\left(cloud\right)}\;<\;vpd\leq VPD2_{G0,\text{max}}$
$OptDeg{\left(VPD\right)}_{G2N}=\{\begin{array}{c} 0\\ 0.3573e^{2.577vpd}-0.3947e^{-7.396vpd}\\ 1\\ -0.004572e^{0.7733vpd}+1.459e^{-0.5429vpd} \end{array}$	$vpd\langle VPD2_{G0,min}\;and\;vpd\rangle VPD2_{G0,max}$ $VPD2_{G0,min\;}\;\leq \;vpd<VPD2_{G1,min\left(nigth\right)}$ $VPD2_{G1,\text{min}\left(nigth\right)\;}\;\leq \;vpd\leq VPD2_{G1,max\left(nigth\right)}$ $VPD2_{G1,max\left(nigth\right)}\;<\;vpd\leq VPD2_{G0,\text{max}}$
$OptDeg{\left(VPD\right)}_{G3S}=\{\begin{array}{c} 0\\ 0.626e^{0.8509vpd}-0.6655e^{-4.974vpd}\\ 1\\ -0.03852e^{0.3585vpd}+1.864e^{-0.3953vpd} \end{array}$	$vpd\langle VPD3_{G0,min}\;and\;vpd\rangle VPD3_{G0,max}$ $VPD3_{G0,min\;}\;\leq \;vpd<VPD3_{G1,min\left(sun\right)}$ $VPD3_{G1,min\left(sun\right)\;}\;\leq \;vpd\leq VPD3_{G1,max\left(sun\right)}$ $VPD3_{G1,max\left(sun\right)}\;<\;vpd\leq VPD3_{G0,\text{max}}$
$OptDeg{\left(VPD\right)}_{G3C}=\{\begin{array}{c} 0\\ 0.6922e^{-4.889vpd}-0.6492e^{0.92124vpd}\\ 1\\ -0.01806e^{0.4577vpd}+1.711e^{-0.4284vpd} \end{array}$	$vpd\langle VPD3_{G0,min}\;and\;vpd\rangle VPD3_{G0,max}$ $VPD3_{G0,min\;}\;\leq \;vpd<VPD3_{G1,min\left(cloud\right)}$ $VPD3_{G1,min\left(cloud\right)\;}\;\leq \;vpd\leq VPD3_{G1,max\left(cloud\right)}$ $VPD3_{G1,max\left(cloud\right)}\;<\;vpd\leq VPD3_{G0,\text{max}}$
$OptDeg{\left(VPD\right)}_{G3N}=\{\begin{array}{c} 0\\ 0.3573e^{2.577vpd}-0.3947e^{-7.396vpd}\\ 1\\ -0.005992e^{0.6209vpd}+1.546e^{-0.4643vpd} \end{array}$	$vpd\langle VPD3_{G0,min}\;and\;vpd\rangle VPD3_{G0,max}$ $VPD3_{G0,min\;}\;\leq \;vpd<VPD3_{G1,min\left(night\right)}$ $VPD3_{G1,min\left(night\right)\;}\;\leq \;vpd\leq VPD3_{G1,max\left(night\right)}$ $VPD3_{G1,max\left(night\right)}\;<\;vpd\leq VPD3_{G0,\text{max}}$

**Table 4:** Descriptive statistics summary of the air temperature, relative humidity and vapor pressure deficit in greenhouse and air temperature, relative humidity of surrounding environment. S.D.: Standard deviation.

Date	Parameter		Daytime	S.D.	Night	S.D.	Average	S.D.	Min	Max	Range
1/10/2018	T (°C)	In	21.6	3.1	16.1	1.5	18.5	3.6	13.9	27.7	13.8
		Out	7.1	5.2	−2.4	2.4	1.7	6.1	−4.9	12	16.9
	RH (%)	In	66.4	8.9	74.4	1.2	70.9	7.2	50.7	79.8	29.1
		Out	43.4	14.3	71	4.6	59.1	17	26.7	75.2	48.5
	VPD	In	0.9	0.31	0.48	0.04	0.66	0.29	0.4	1.38	0.99
1/17/2018	T (°C)	In	19	1.9	18.1	1.6	18.5	1.8	14.4	23.2	8.8
		Out	7.2	3.9	−0.5	3.1	2.9	5.1	−4.6	10.5	15.1
	RH (%)	In	65.7	11.9	73	1.1	69.8	8.7	46.6	82.6	36
		Out	41.3	12.2	66.1	8.3	55.3	16	30.3	76.3	46
	VPD	In	0.77	0.28	0.58	0.06	0.66	0.22	0.36	1.26	0.9
6/10/2018	T (°C)	In	29.1	8.1	14.7	2.1	23.5	9.6	11.8	38	26.2
		Out	27.5	6.8	17	3	23.5	7.6	12.3	39	26.7
	RH (%)	In	45.1	20.2	90.3	6.7	62.6	27.5	27.5	96	68.5
		Out	38.1	15.8	61.7	8.7	47.3	17.8	23.5	74	50.5
	VPD	In	2.73	1.49	0.18	0.17	1.74	1.7	0.06	4.72	4.66
7/29/2018	T (°C)	In	38.8	9	24.7	3	33.4	10	19.6	51.2	31.6
		Out	36.8	6.9	27.2	3.2	33.1	7.5	21.2	50.5	29.3
	RH (%)	In	19.3	9.5	37.3	5.1	26.2	11.9	10.2	46.2	36
		Out	18.9	6.1	23	4.9	22	6.9	11.2	38	26.8
	VPD	In	6.4	3.03	2.01	0.54	4.72	3.22	1.23	11.76	10.53
